# Supplementary material for: Constituents Isolated from the Leaves of Glycyrrhiza uralansis and Their Anti-Inflammatory Activities on LPS-Induced RAW264.7 Cells
Source: Molecules. 2019 May 18;24(10):1923. doi: 10.3390/molecules24101923 (PMC6571819; doi:10.3390/molecules24101923)
Supplement: Supplementary file 1 [file molecules-24-01923-s001.pdf]

SUPPLEMENTAYR MATERIALS

Constituents isolated from the leaves of *Glycyrrhiza uralansis* and their anti-inflammatory activities on LPS-induced RAW264.7 cells

Liyao Wang [1], Kaixue Zhang [1], Shu Han [2], Liu Zhang [1], Haiying Bai [1], Fang Bao [1], Yan Zeng [3], Jiyong Wang [3], Hong Du [2], Yingqian Liu[1], and Zhigang Yang [1,*]

[1] School of Pharmacy, Lanzhou University, LanZhou, China; lywang17@lzu.edu.cn(L.Y.W.); zhangkx2018@lzu.edu.cn(K.X.Z.); 859842580@qq.com(L.Z.); baihy14@lzu.edu.cn(H.Y.B.); baof16@lzu.edu.cn(F.B.); yqliu@lzu.edu.cn(Y.Q.L)

[2] School of Chinese Materia Medica, Beijing University of Chinese Medicine, Beijing, China; hs361015@163.com(S.H.); duhong@vip.163.com(H.D.)

[3] China National Traditional Chinese Medicine Co.,Ltd., Beijing, China; wangjiyong75@163.com(J.Y.W.); zyzy1221@126.com(Y.Z.)

* Correspondence: yangzg@lzu.edu.cn(Z.G.Y.); Tel.: +86-931-8915202

Abstract: Licorice, the root and rhizome of *Glycyrrhiza uralansis* Fisch, is one of the most frequently used Traditional Chinese Medicines in rigorous clinical trials to remove toxins, sputum and relieve coughing. However, the aerial parts are not used so widely at present. It has been reported that the aerial parts have many bioactivities such as anti-microbial and anti-HIV activities. In this study, we aimed to discover the bioactive compounds from the leaves of *G. uralensis*. Four new compounds, licostilbene A-B (**1-2**) and licofuranol A-B (**3-4**), together with eight known flavonoids (**5-12**) were isolated and identified from the leaves of *G. uralensis*. Their structures were elucidated mainly by the interpretation of high-resolution electrospray mass spectrometry (HR-ESI-MS) and nuclear magnetic resonance (NMR) spectroscopic data. Compared with quercetin, which showed a 50% inhibitory concentration (IC_{50}) value of 4.08 μg/ml, compounds **1-9** showed significant anti-inflammatory activities by inhibiting lipopolysaccharide (LPS)-induced nitric oxide (NO) production with IC_{50} values of 2.60, 2.15, 3.21, 3.25, 2.00, 3.45, 2.53, 3.13 and 3.17 μg/ml, respectively. The discovery of these active compounds is important for the prevention and treatment of inflammation.

Keywords: *Glycyrrhiza uralensis*; licostilbene; licofuranol; anti-inflammatory activity

Figures	Pages
**Figure S1**: The key HMBC correlations observed for compounds **1-4**	1
**Figure S2**: UV spectrum of compound **1**	1
**Figure S3**:HR-ESI-MS positive mode ion of compound **1**	2
**Figure S4**: ^{1}H-NMR spectrum of compound **1**	2
**Figure S5**: ^{13}C-NMR spectrum of compound **1**	3

**Figure S6**: DEPT 135 spectrum of compound **1**	3
Figure **S7**: HSQC spectrum of compound **1**	4
**Figure S8**: HMBC spectrum of compound **1**	4
**Figure S9**: NOESY spectrum of compound **1**	5
**Figure S10**: COSY spectrum of compound **1**	5
**Figure S11**: UV spectrum of compound **2**	6
**Figure S12**: HR-ESI-MS positive mode ion of compound **2**	6
**Figure S13**: ^{1}H-NMR spectrum of compound **2**	7
**Figure S14**: ^{13}C-NMR spectrum of compound **2**	7
**Figure S15**: DEPT 135 spectrum of compound **2**	8
**Figure S16**: HSQC spectrum of compound **2**	8
**Figure S17**: HMBC spectrum of compound **2**	9
**Figure S18**: NOESY spectrum of compound **2**	9
**Figure S19**: COSY spectrum of compound **2**	10
**Figure S20**: UV spectrum of compound **3**	10
**Figure S21**:HR-ESI-MS positive mode ion of compound **3**	11
**Figure S22**: ^{1}H-NMR spectrum of compound **3**	11
**Figure S23**: ^{13}C-NMR spectrum of compound **3**	12
**Figure S24**: DEPT 135 spectrum of compound **3**	12
**Figure S25**: HSQC spectrum of compound **3**	13
**Figure S26**: HMBC spectrum of compound **3**	13
**Figure S27**: NOESY spectrum of compound **3**	14
**Figure S28**: COSY spectrum of compound **3**	14
**Figure S39**: UV spectrum of compound **4**	15
**Figure S30**:HR-ESI-MS positive mode ion of compound **4**	15
**Figure S31**: ^{1}H-NMR spectrum of compound **4**	16
**Figure S32**: ^{13}C-NMR spectrum of compound **4**	16

**Figure S33**: DEPT 135 spectrum of compound **4**	17
**Figure S34**: HSQC spectrum of compound **4**	17
**Figure S35**: HMBC spectrum of compound **4**	18
**Figure S36**: NOESY spectrum of compound **4**	18
**Figure S37**: COSY spectrum of compound **4**	19
**Figure S38**: ^{1}H-NMR spectrum of compound **5**	19
**Figure S39**: ^{1}H-NMR spectrum of compound **6**	20
**Figure S40**: ^{1}H-NMR spectrum of compound **7**	20
**Figure S41**: ^{1}H-NMR spectrum of compound **8**	21
**Figure S42**: ^{1}H-NMR spectrum of compound **9**	21

**Table**	**Pages**
**Table S1**: The % composition of compounds **1-12** in the leaves of *G. uralensis*	22

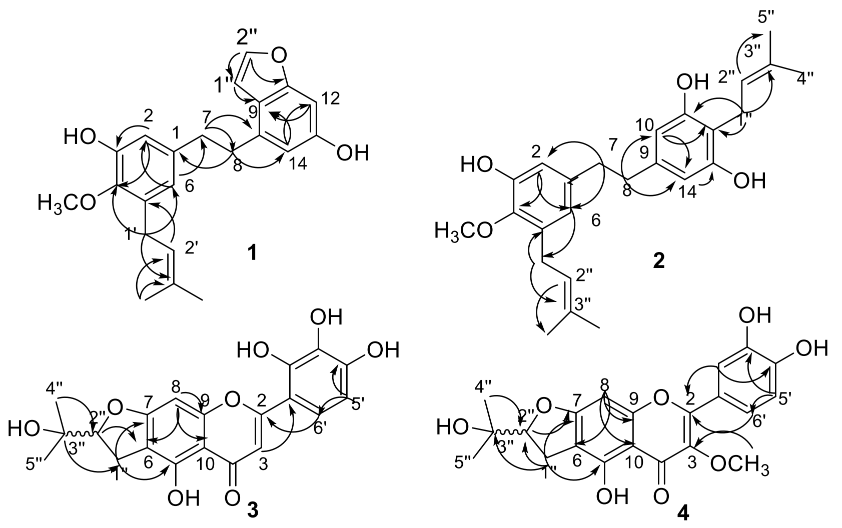

Figure S1: The key HMBC correlations observed for compounds **1**-**4**

Compound **1**

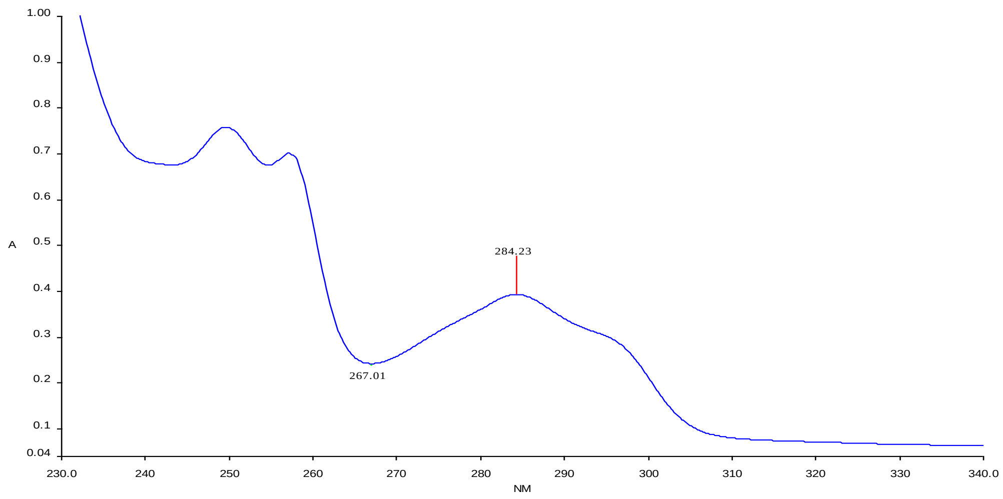

Figure S2: UV spectrum of compound **1** (absorption maxima at 250, 259 and 284 nm)

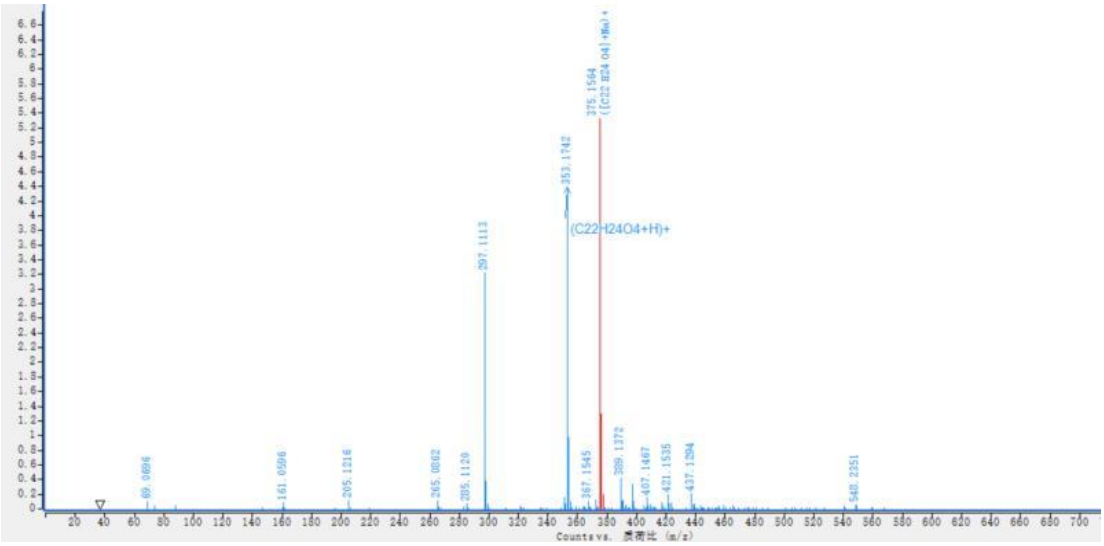

Figure S3: HR-ESI-MS positive mode ion of compound **1**

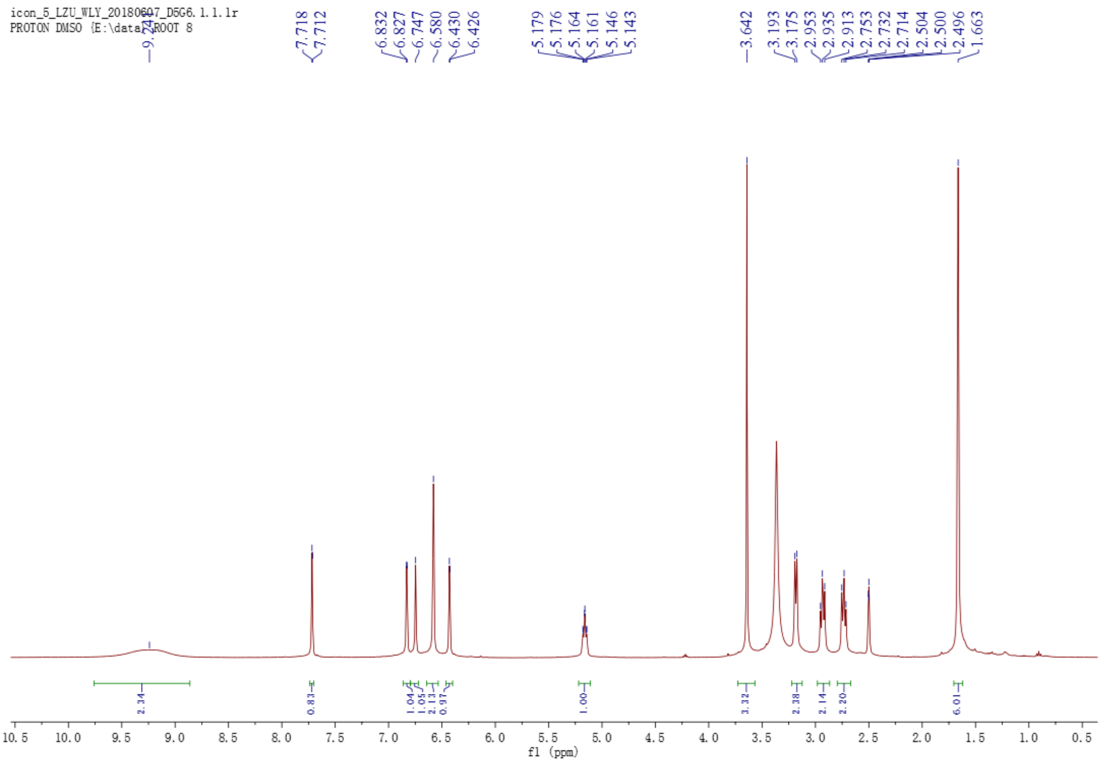

Figure S4: ^{1}H-NMR spectrum of compound **1**

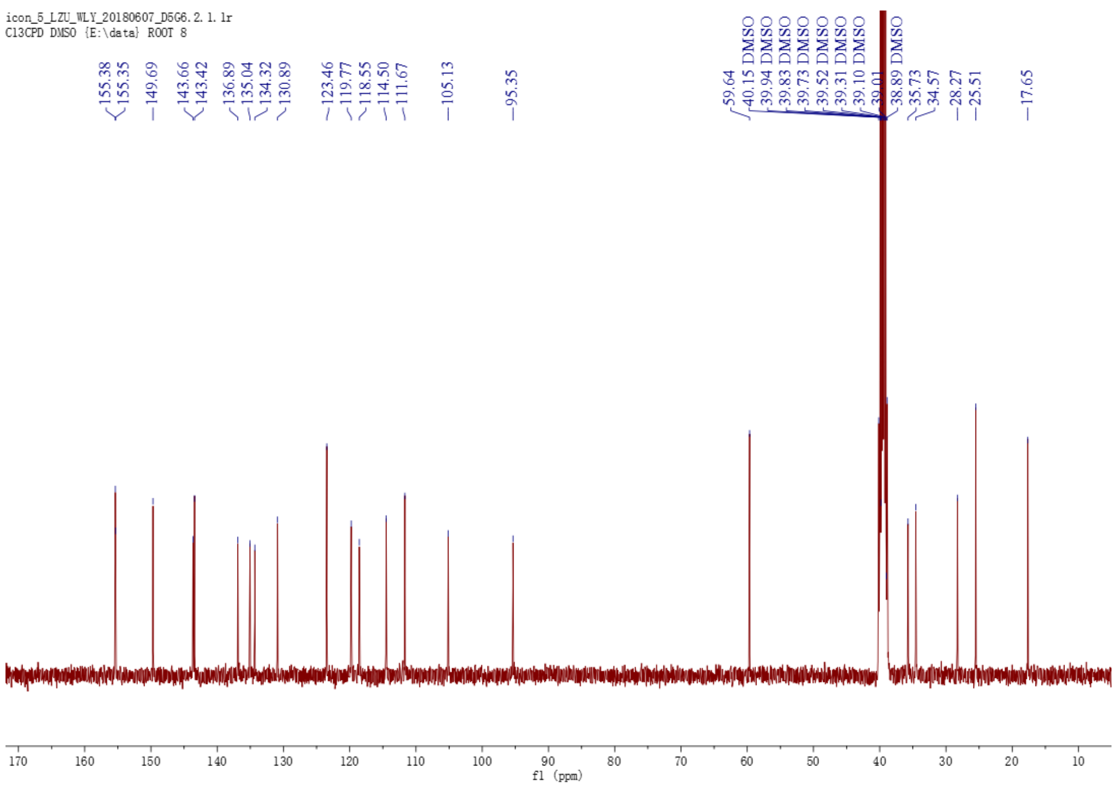

Figure S5: ^{13}C-NMR spectrum of compound **1**

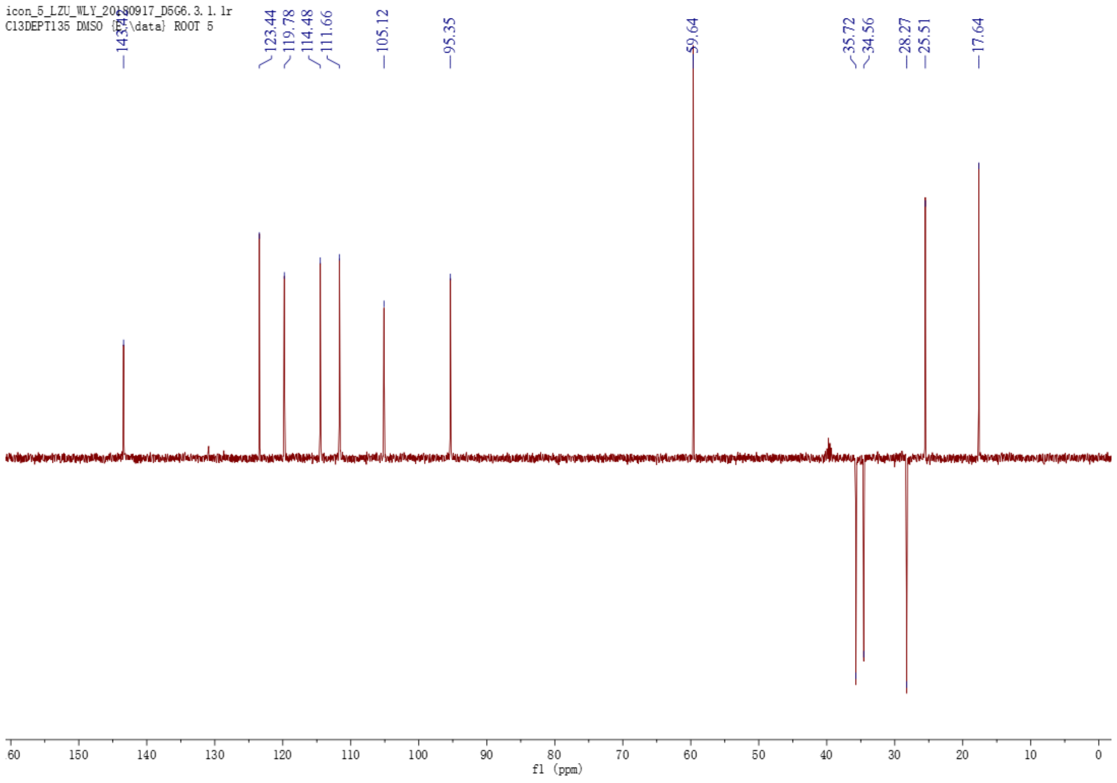

Figure S6: DEPT 135 spectrum of compound **1**

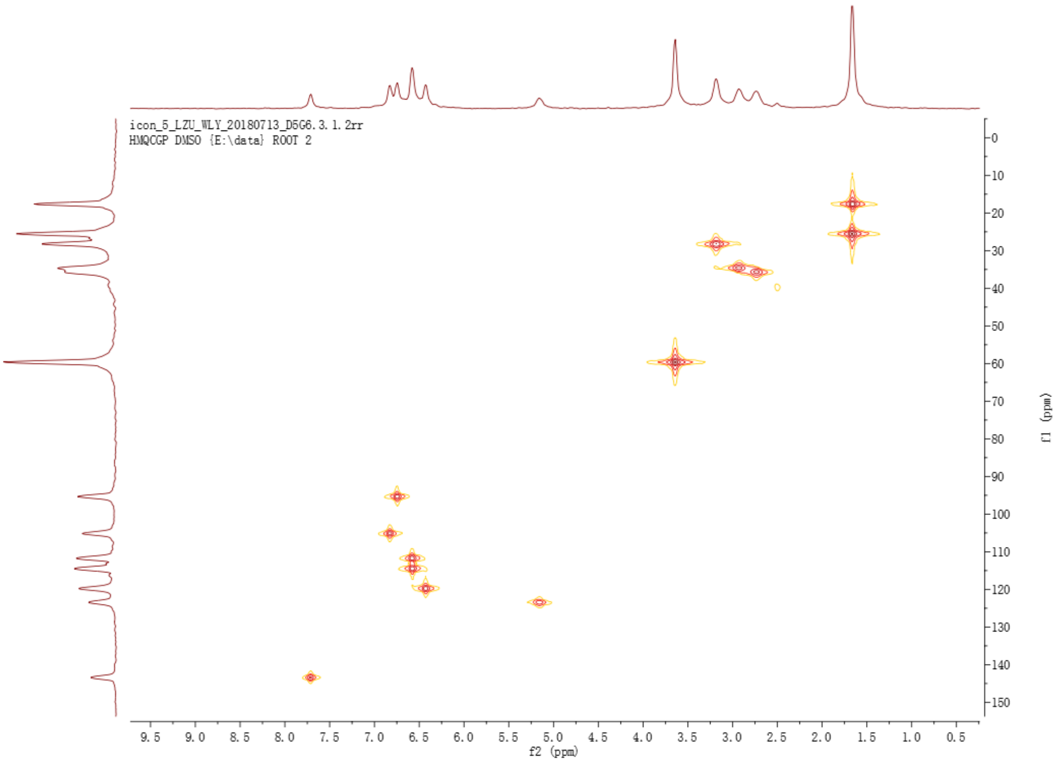

Figure S7: HSQC spectrum of compound **1**

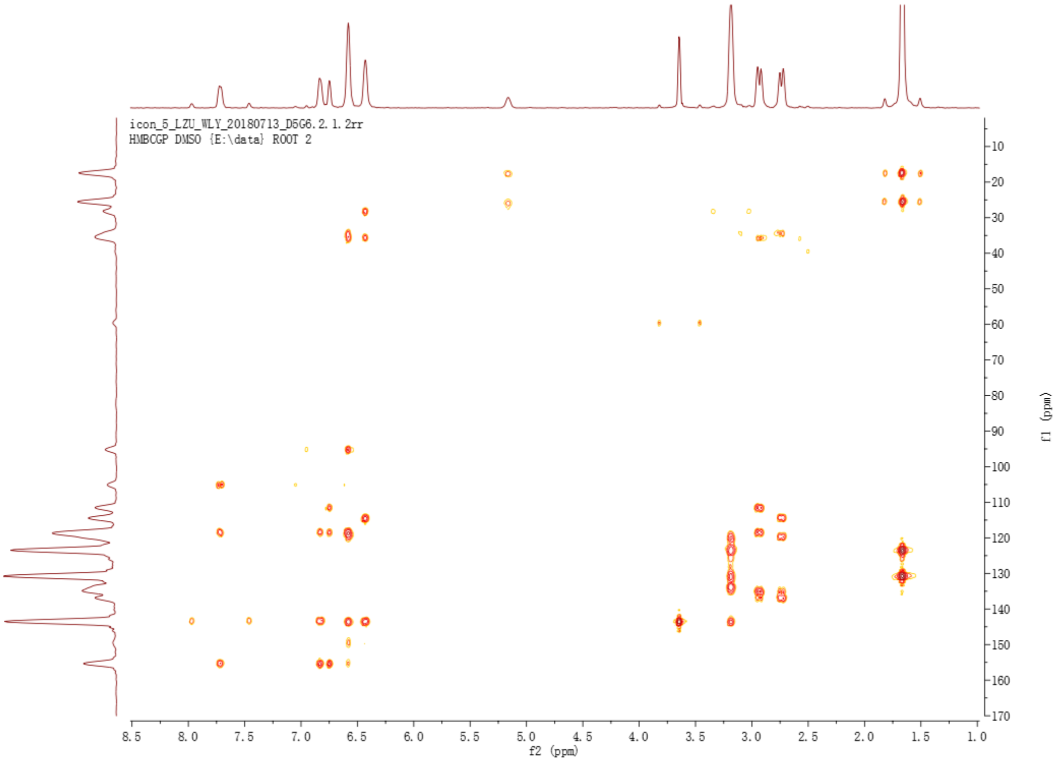

Figure S8: HMBC spectrum of compound **1**

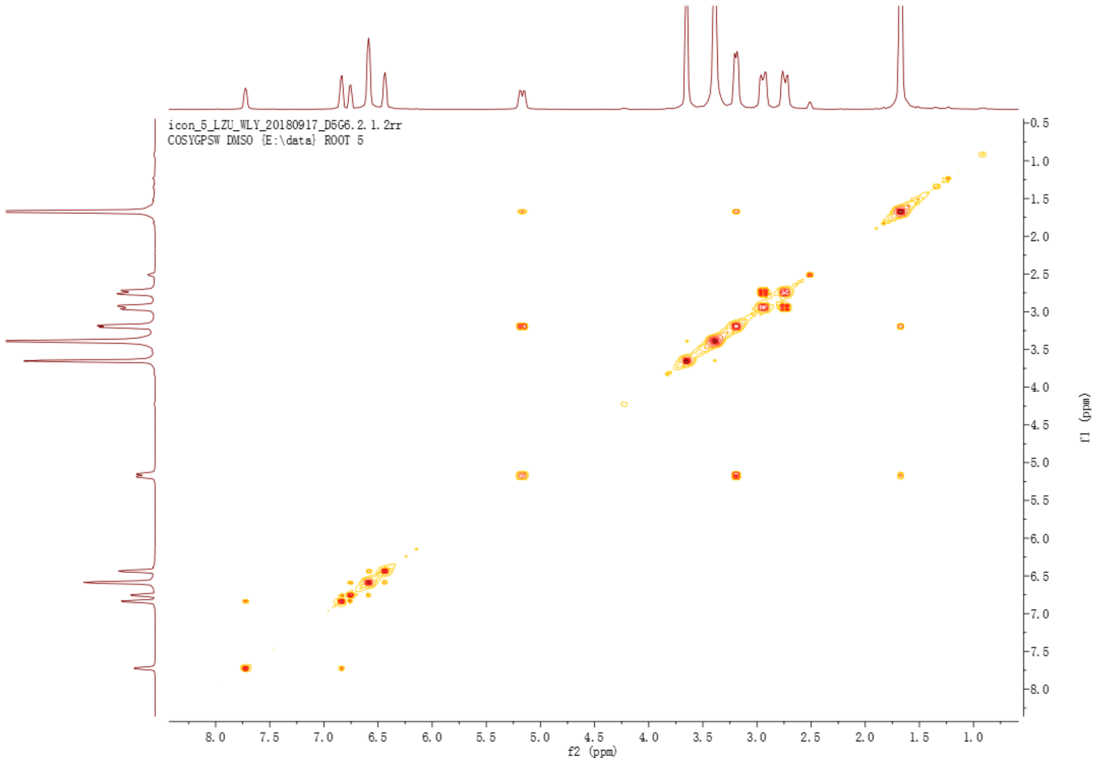

Figure S9: NOESY spectrum of compound **1**

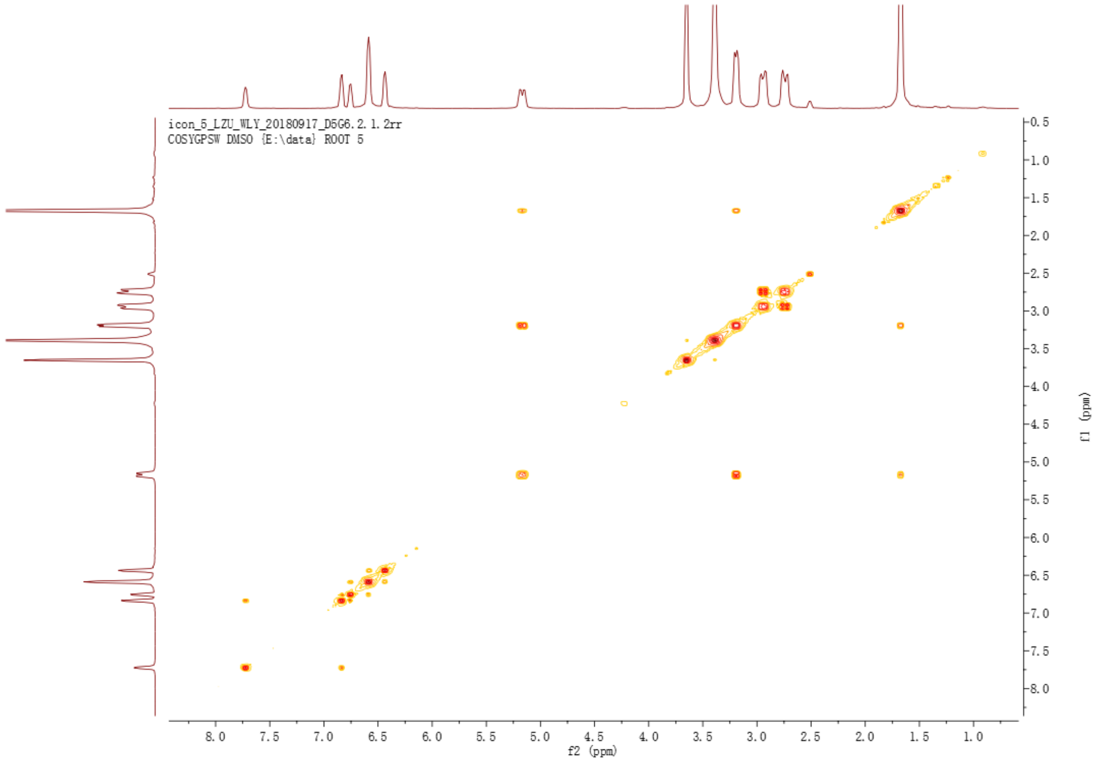

Figure S10: COSY spectrum of compound **1**

Compound **2**

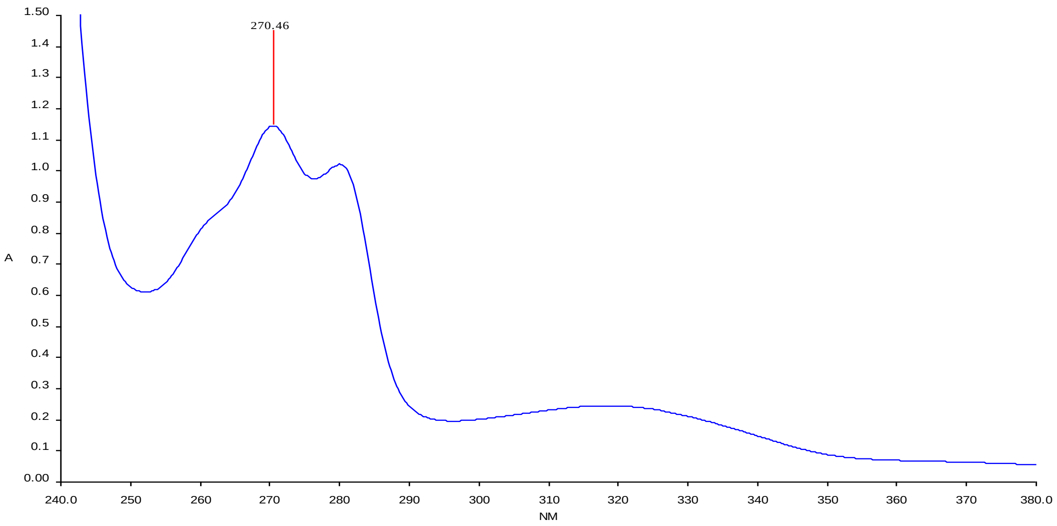

Figure S11: UV spectrum of compound **2** (absorption maxima at 270 and 282 nm)

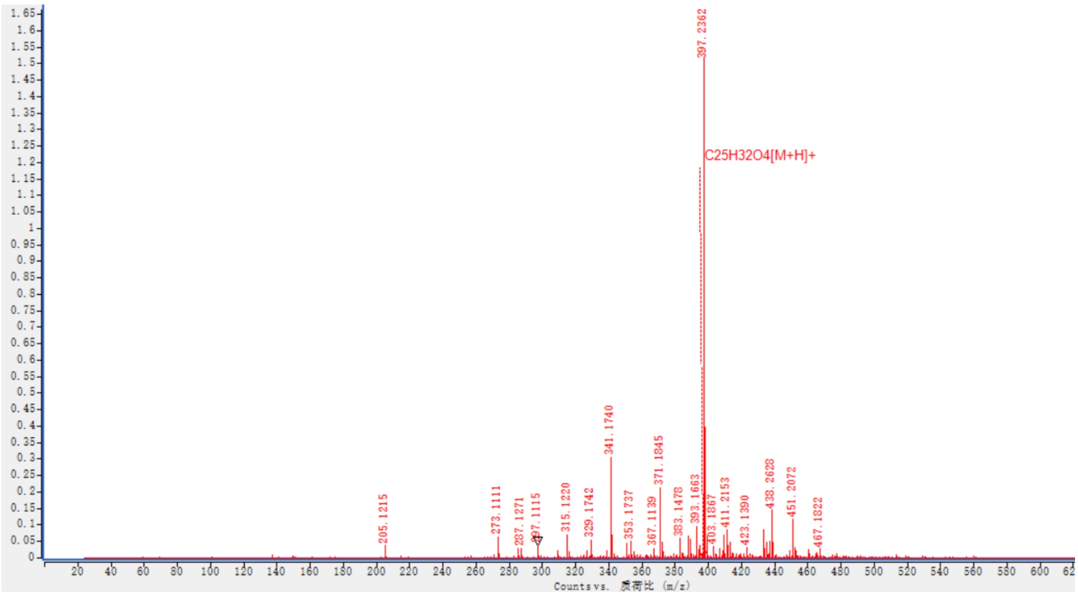

Figure S12: HR-ESI-MS positive mode ion of compound **2**

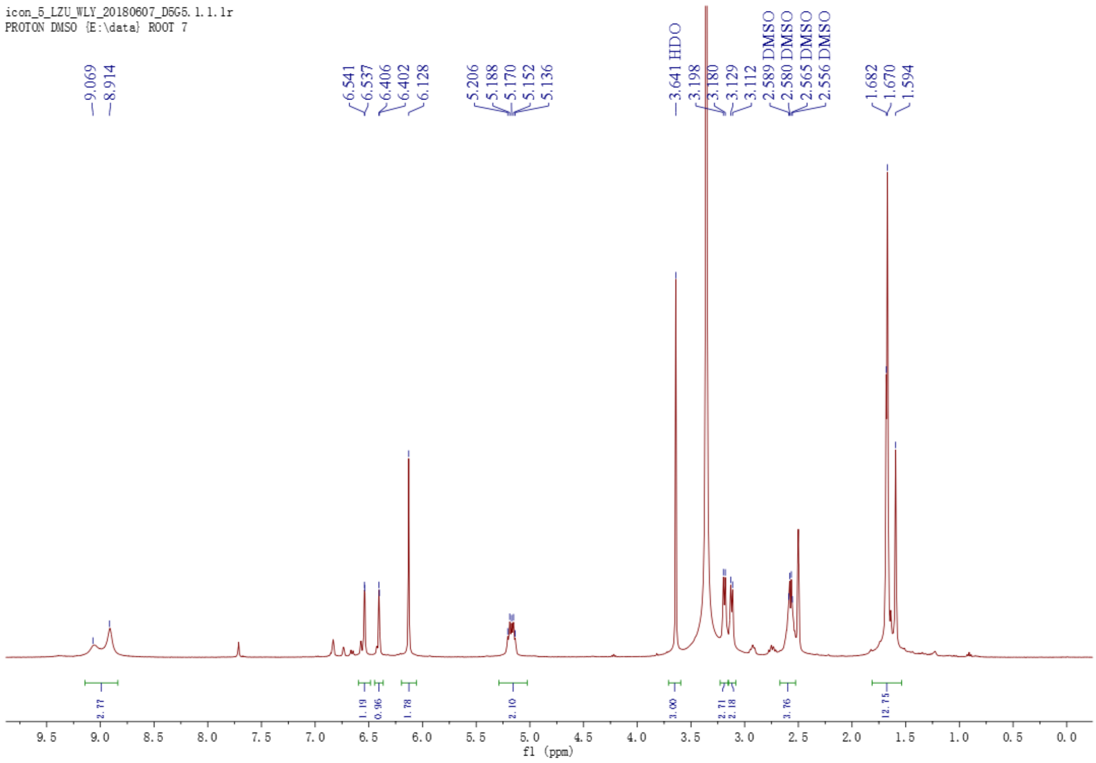

Figure S13: ^{1}H-NMR spectrum of compound **2**

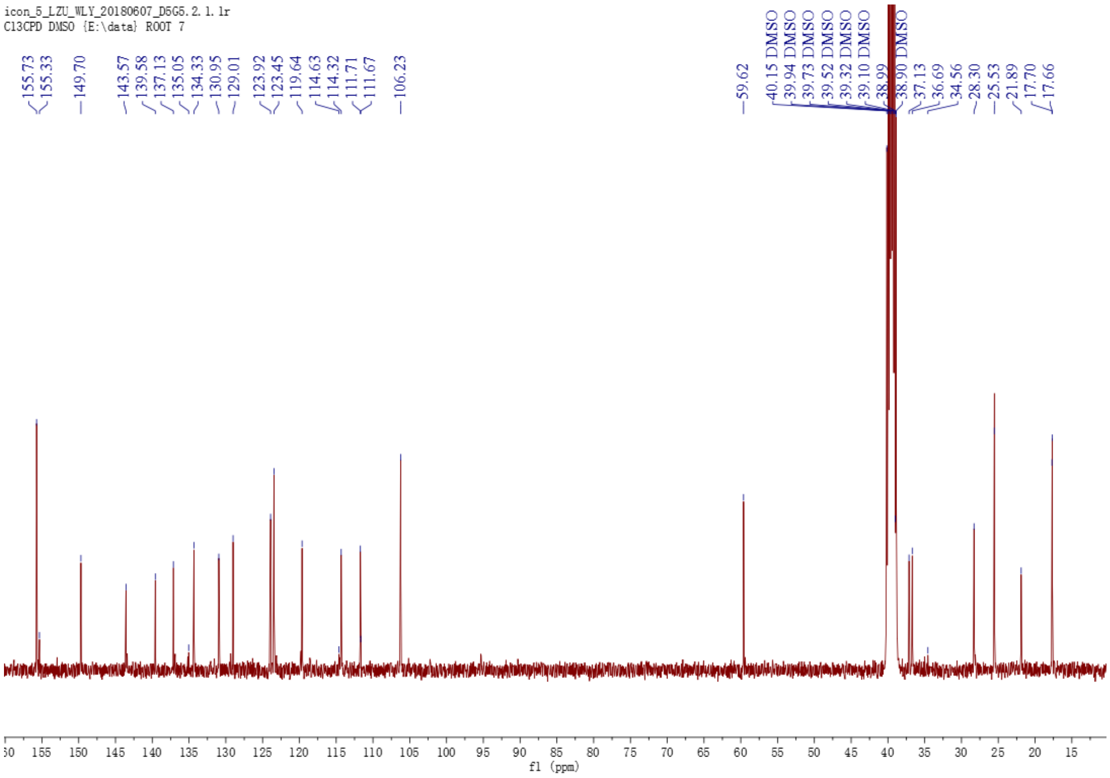

Figure S14: ^{13}C-NMR spectrum of compound **2**

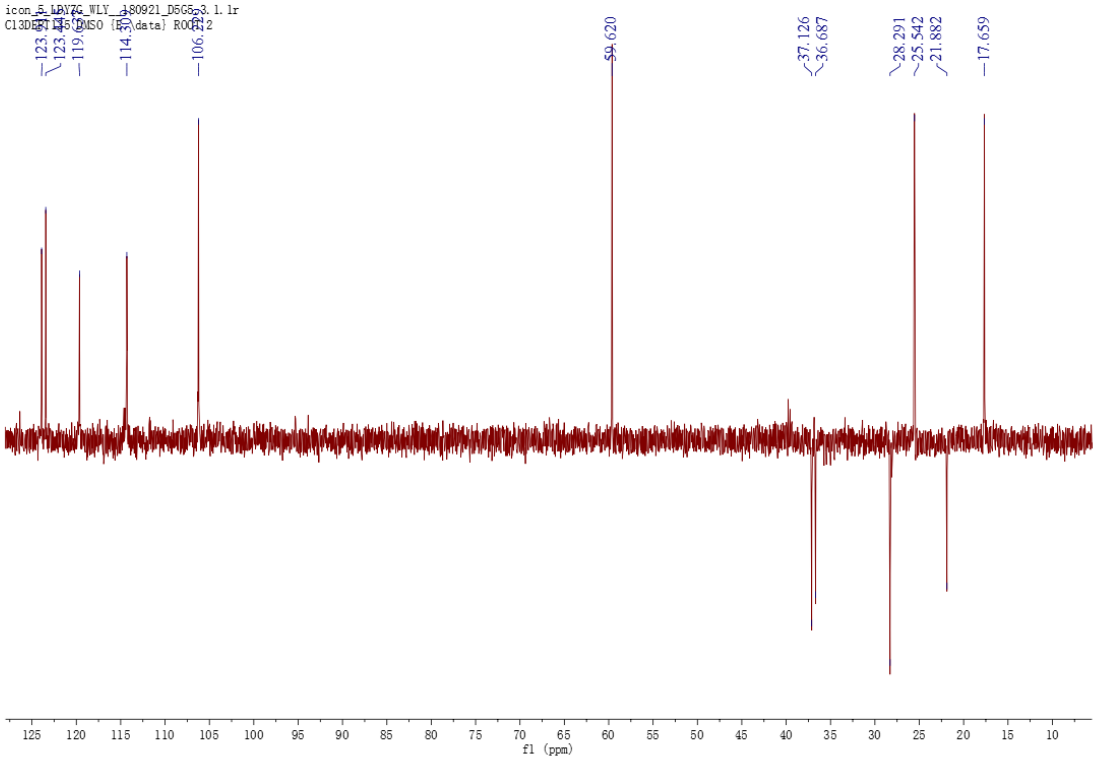

Figure S15: DEPT 135 spectrum of compound **2**

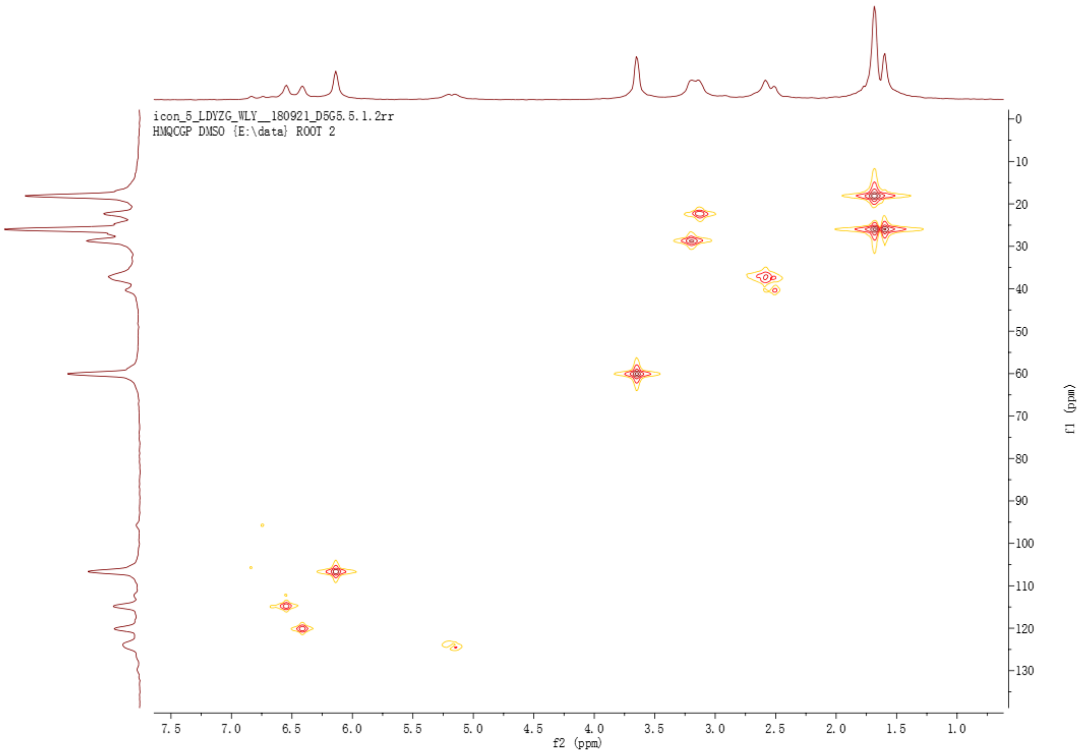

Figure S16: HSQC spectrum of compound **2**

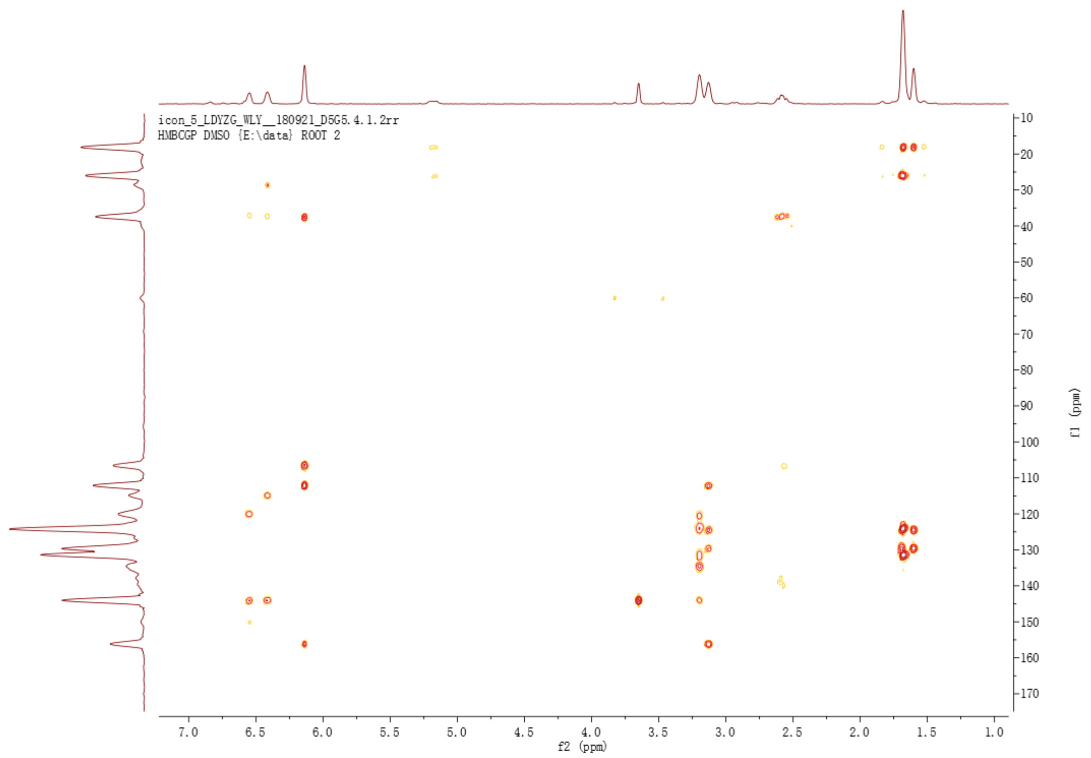

Figure S17: HMBC spectrum of compound **2**

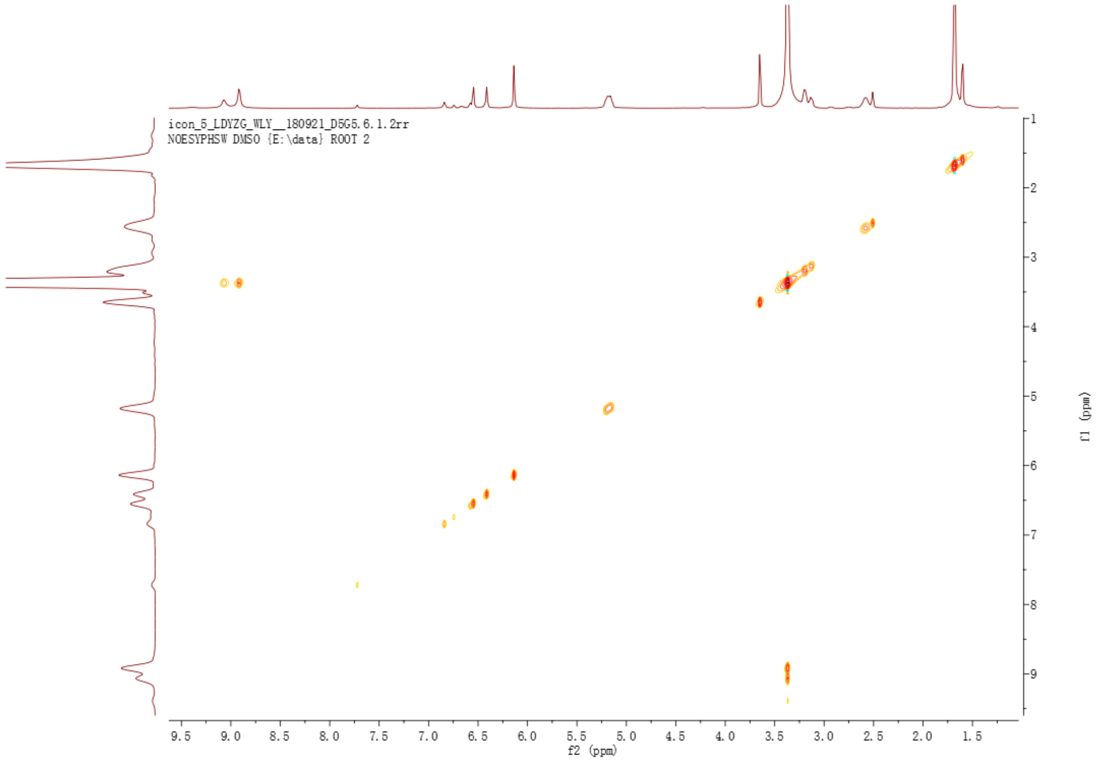

Figure S18: NOESY spectrum of compound **2**

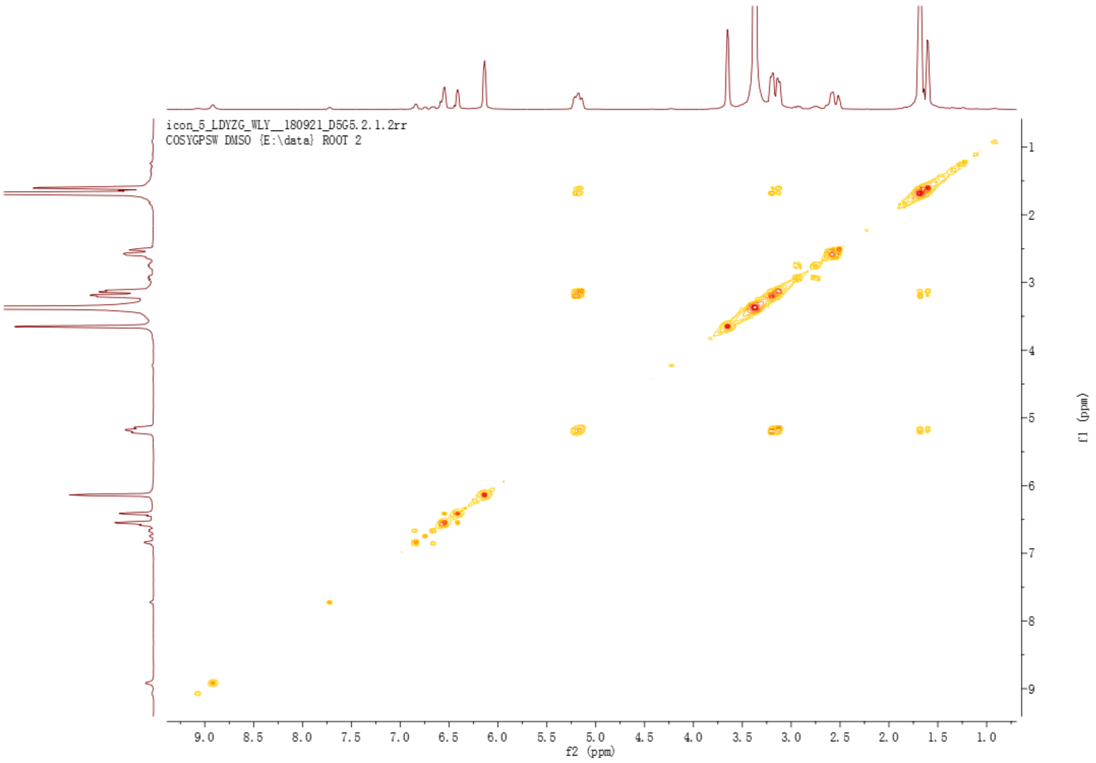

Figure S19: COSY spectrum of compound **2**

Compound**3**

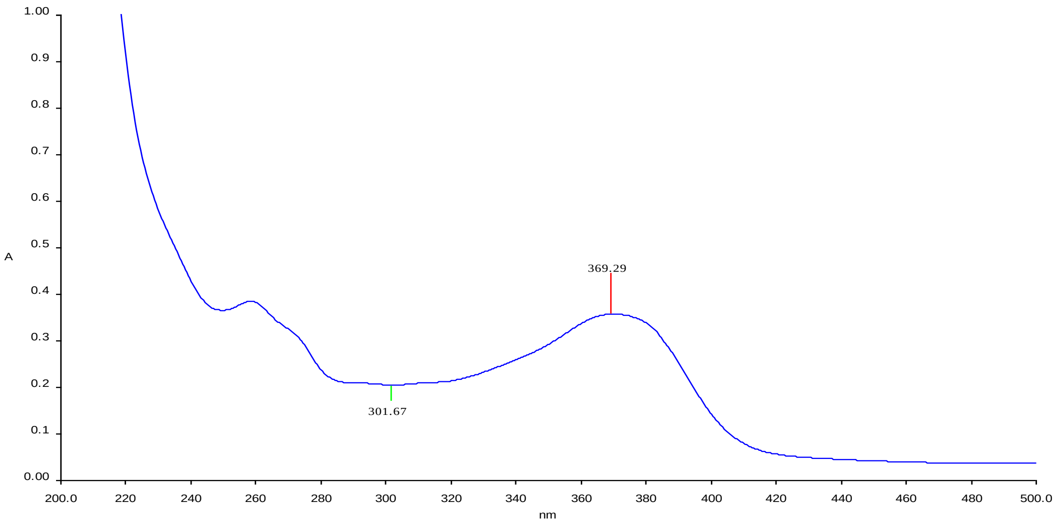

Figure S20: UV spectrum of compound **3** (absorption maxima at 258 and 369 nm)

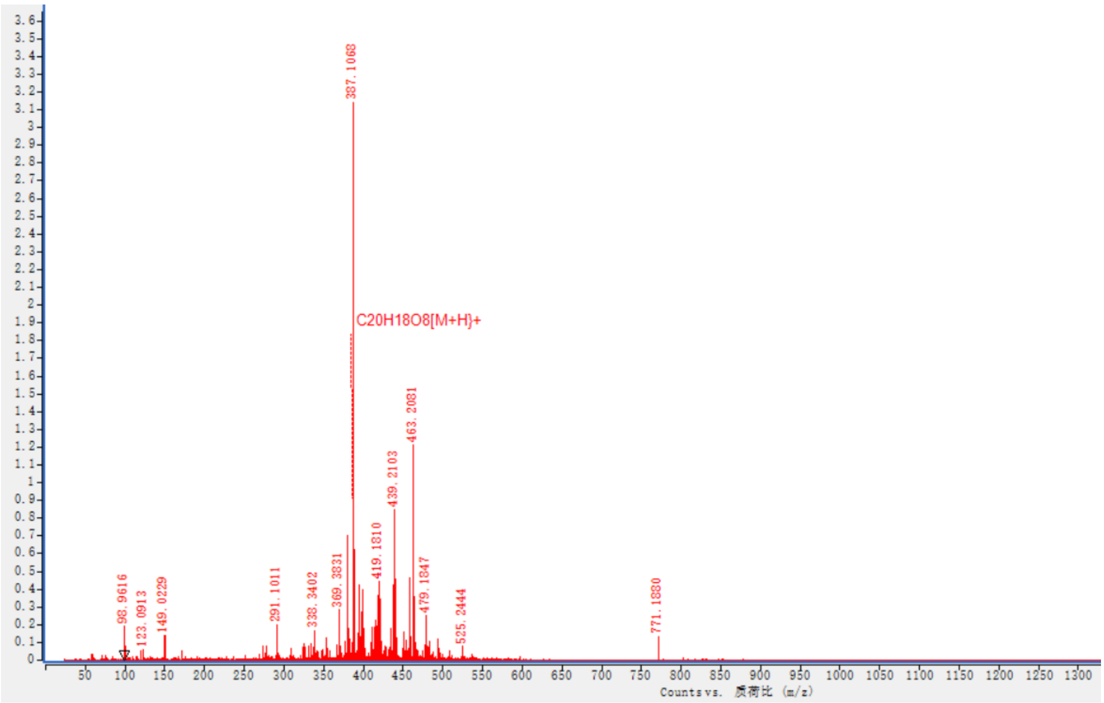

Figure S21:HR-ESI-MS positive mode ion of compound **3**

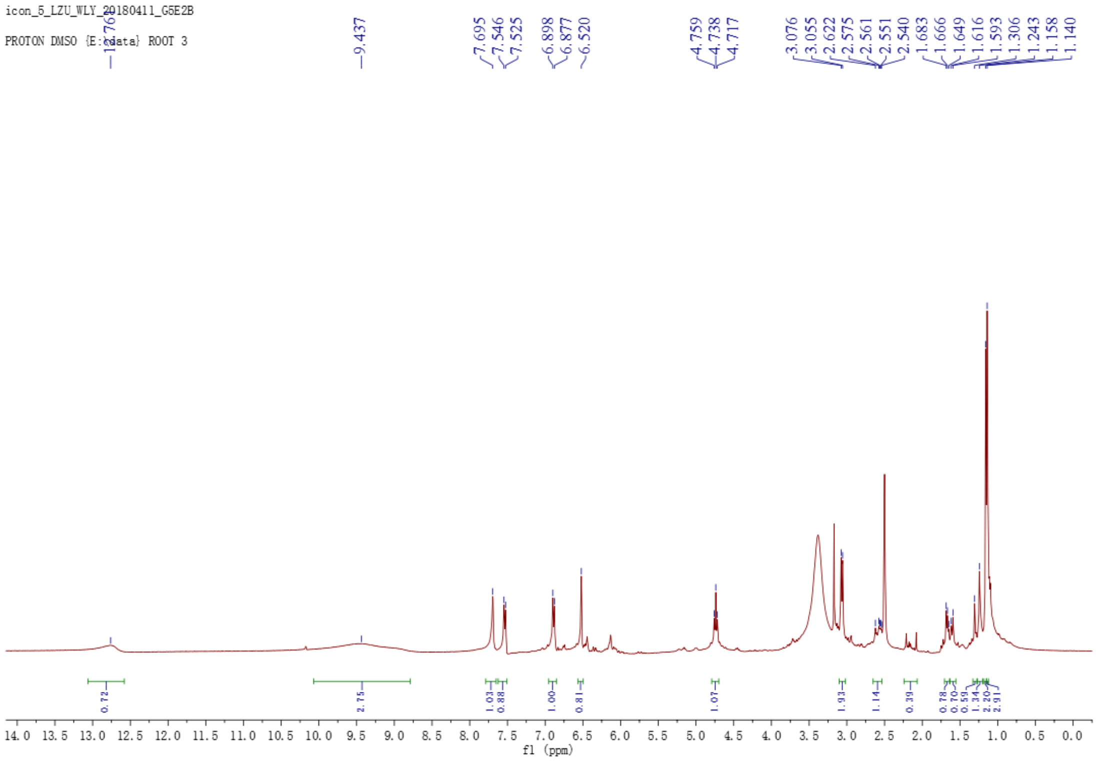

Figure S22: ^{1}H-NMR spectrum of compound **3**

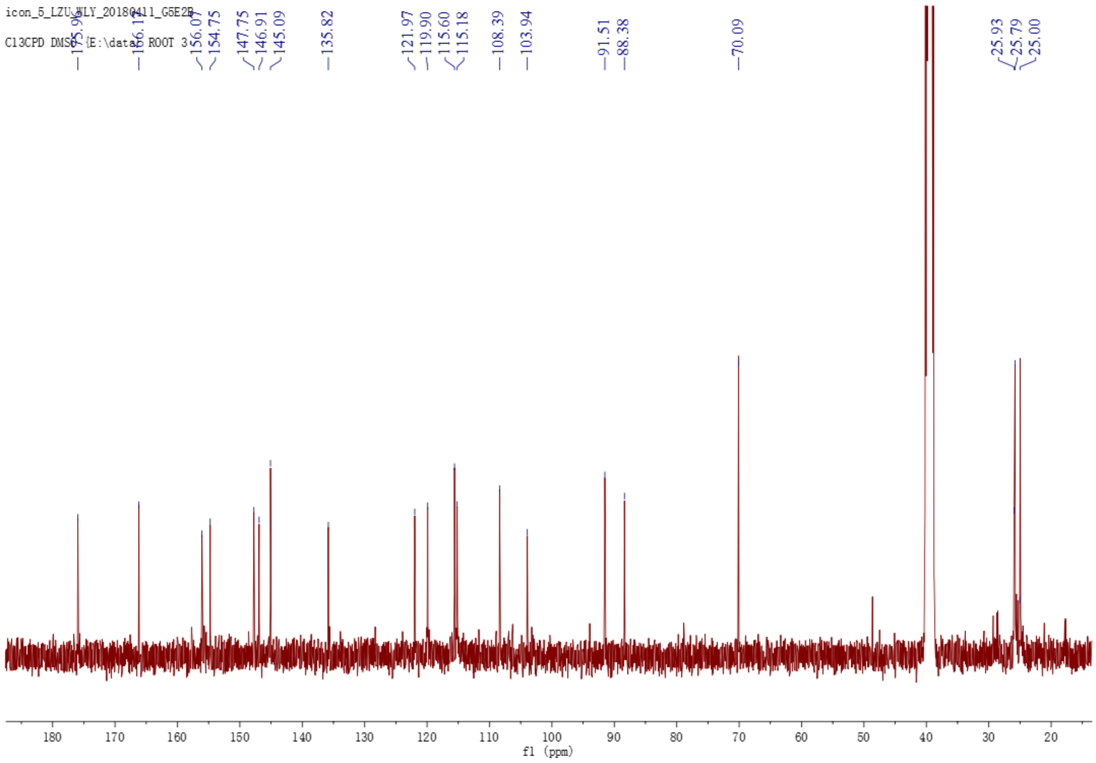

Figure S23: ^{13}C-NMR spectrum of compound **3**

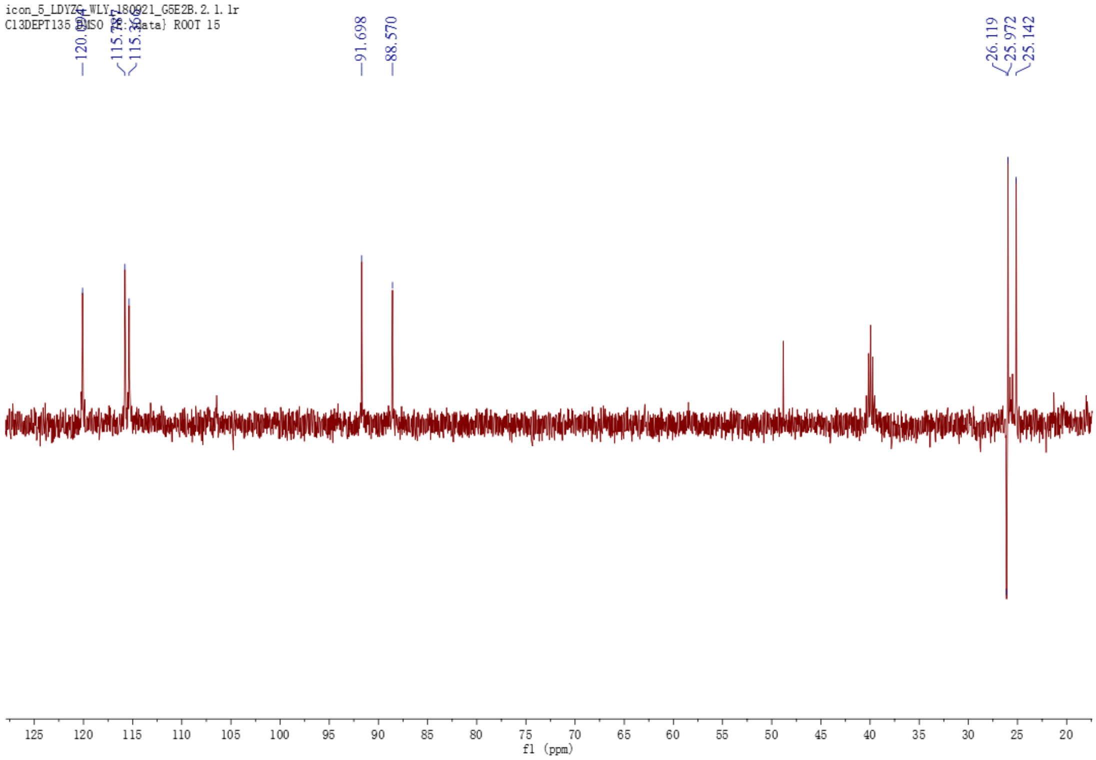

Figure S24: DEPT 135 spectrum of compound **3**

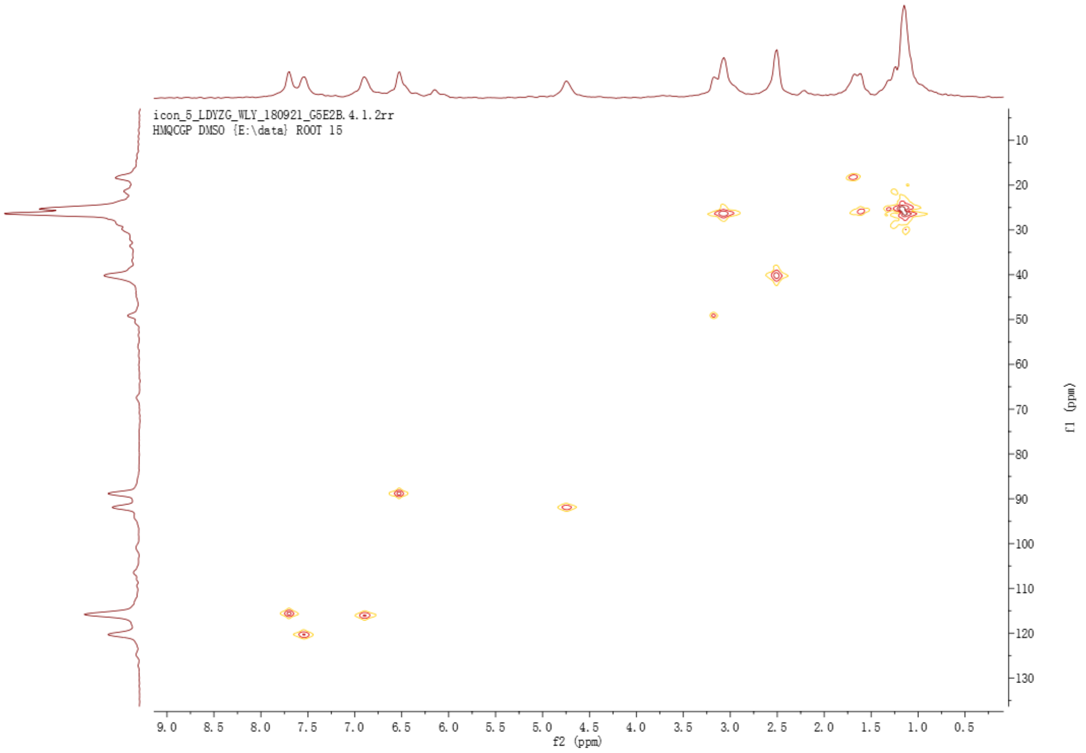

Figure S25: HSQC spectrum of compound **3**

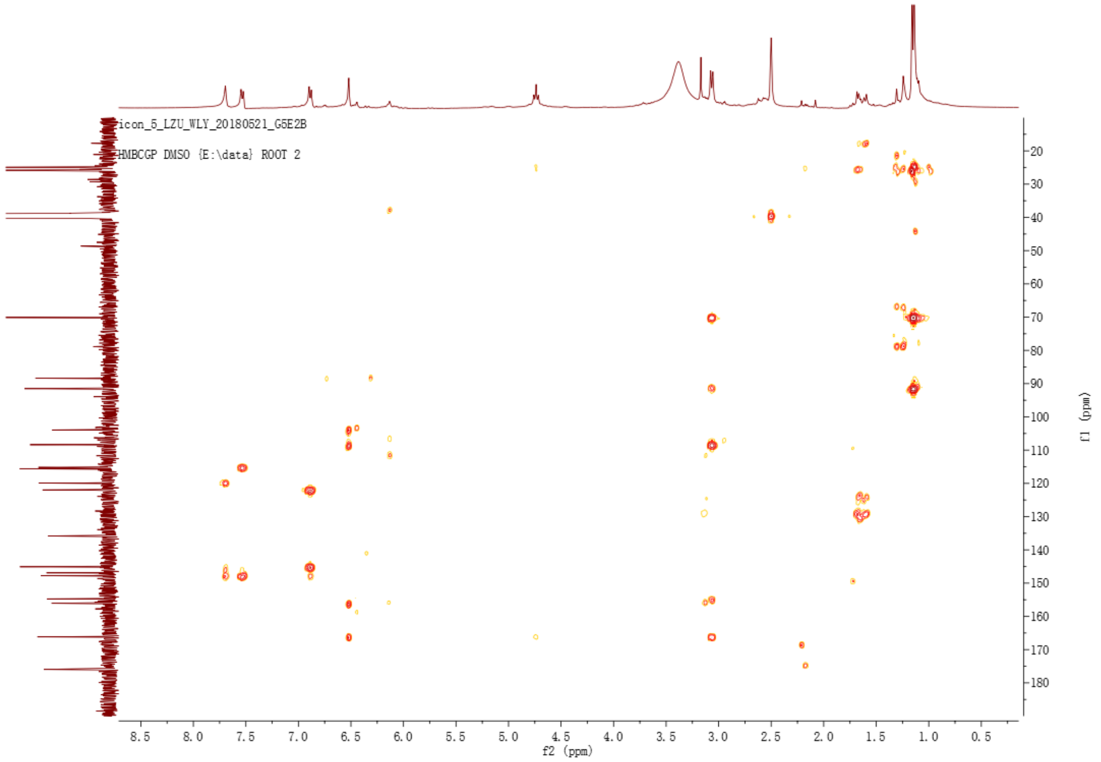

Figure S26: HMBC spectrum of compound **3**

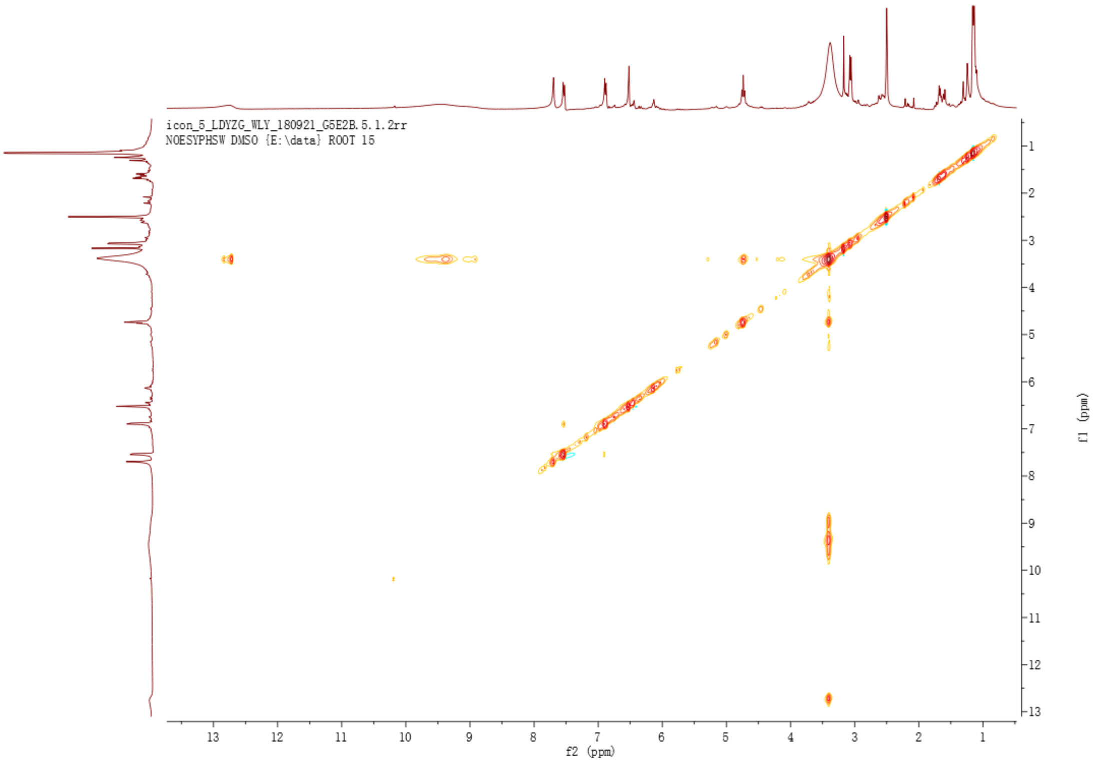

Figure S27: NOESY spectrum of compound **3**

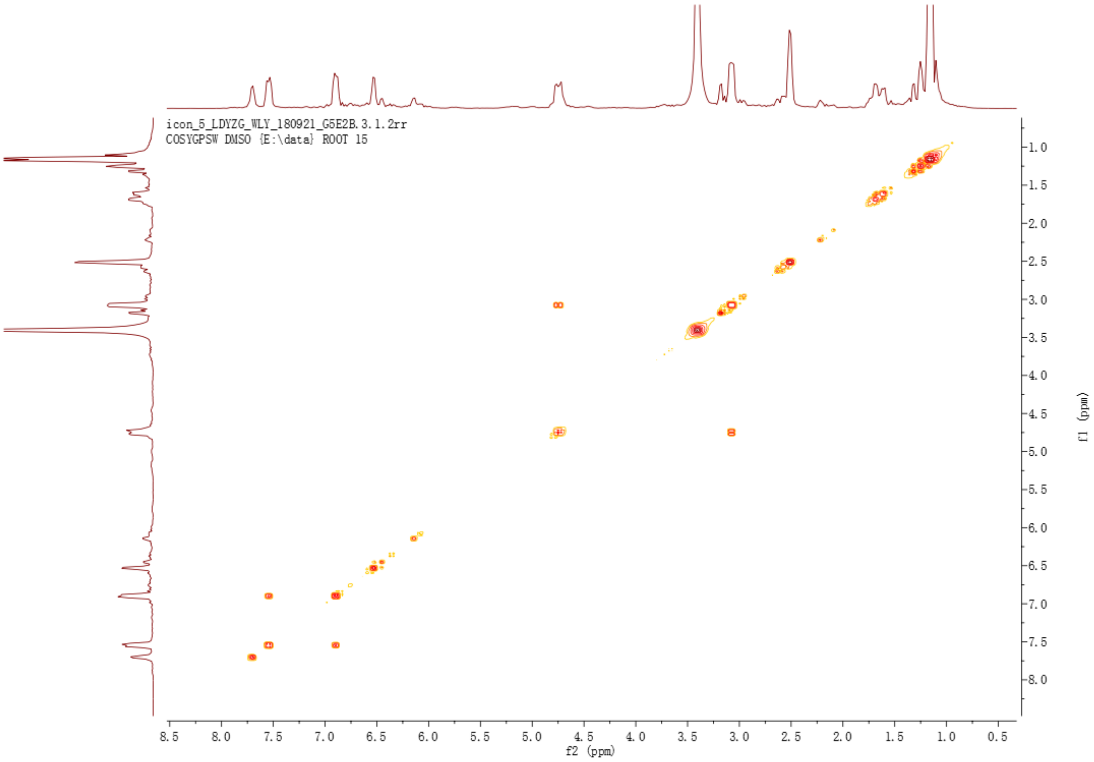

Figure S28: COSY spectrum of compound **3**

Compound **4**

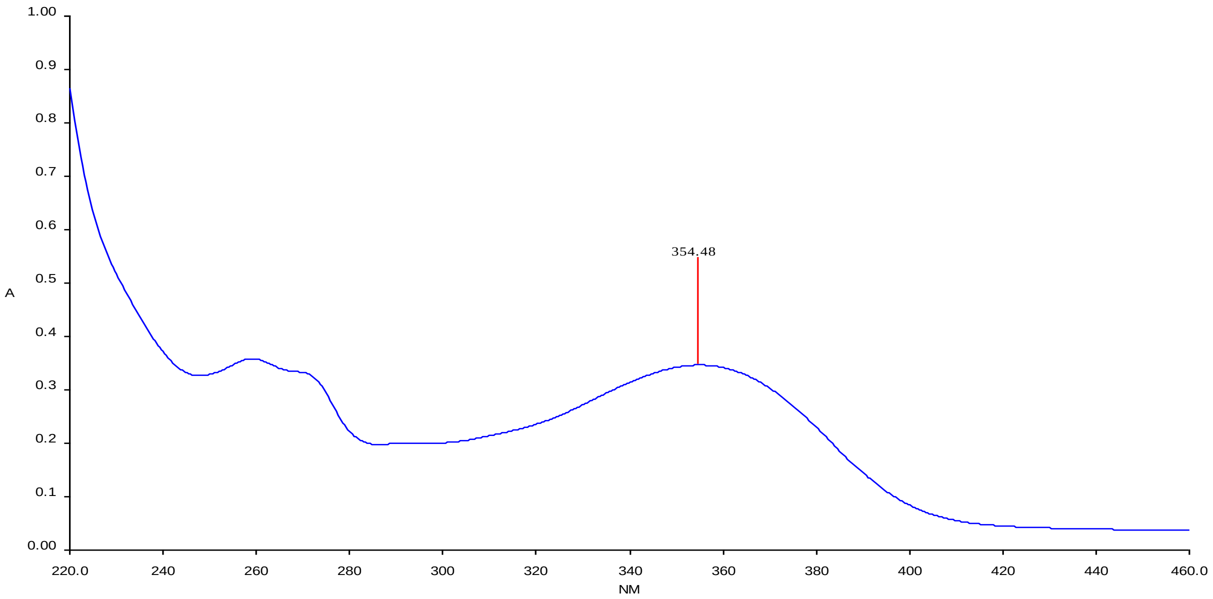

Figure S29: UV spectrum of compound **4** (absorption maxima at 259 and 354 nm)

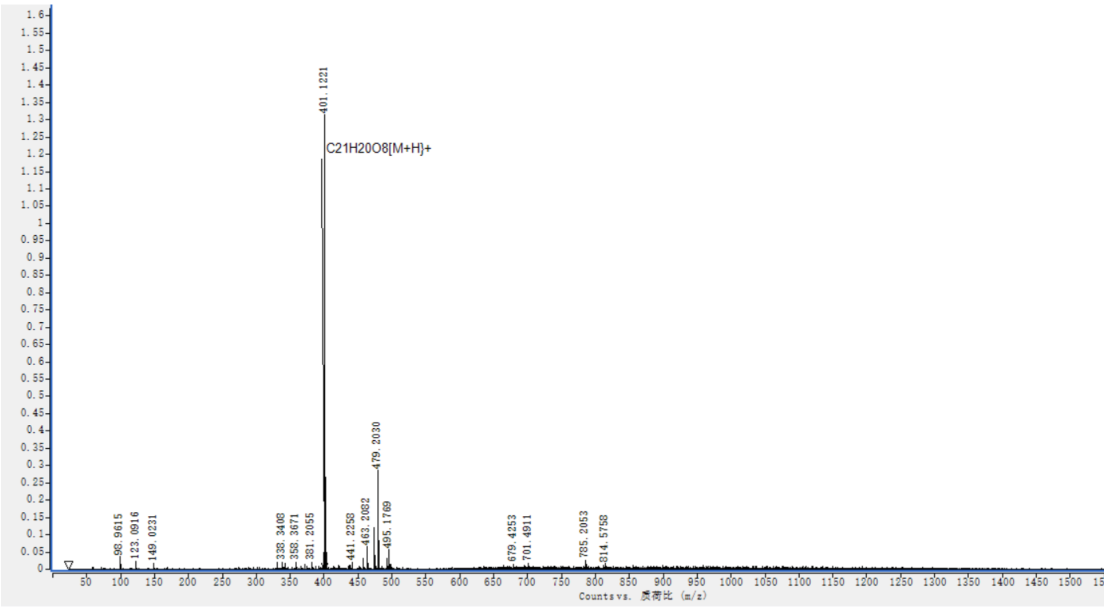

Figure S30: HR-ESI-MS positive mode ion of compound **4**

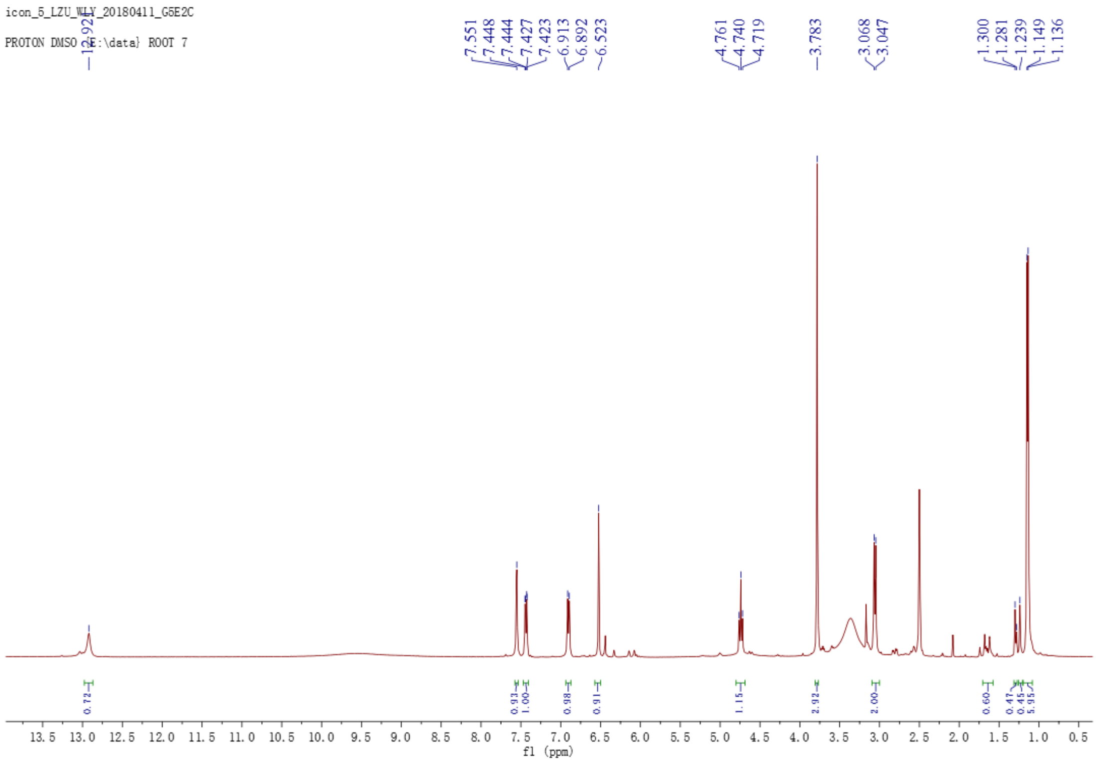

Figure S31: ^{1}H-NMR spectrum of compound **4**

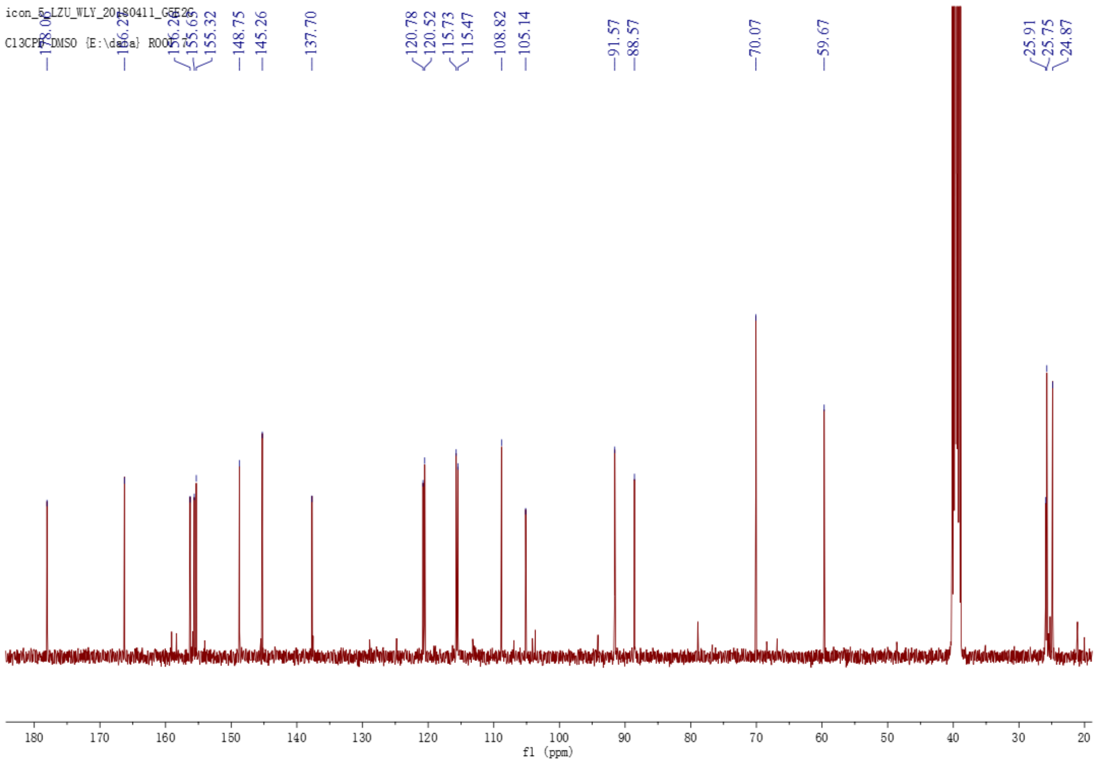

Figure S32: ^{13}C-NMR spectrum of compound **4**

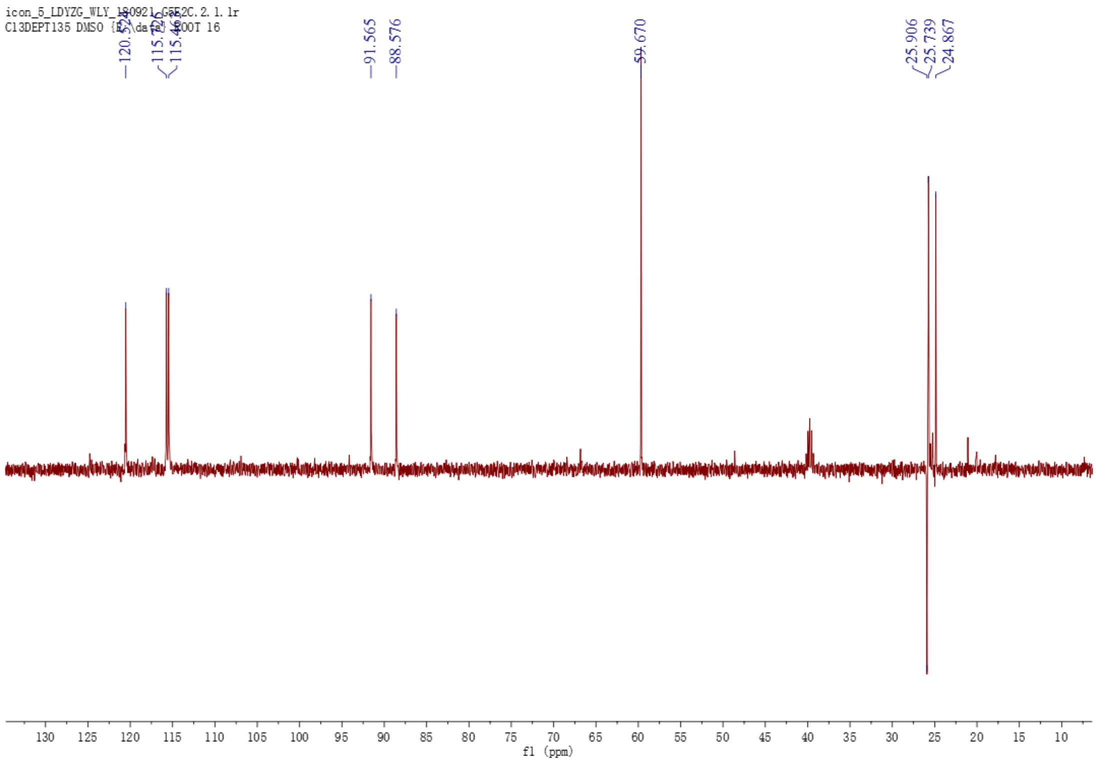

Figure S33: DEPT 135 spectrum of compound **4**

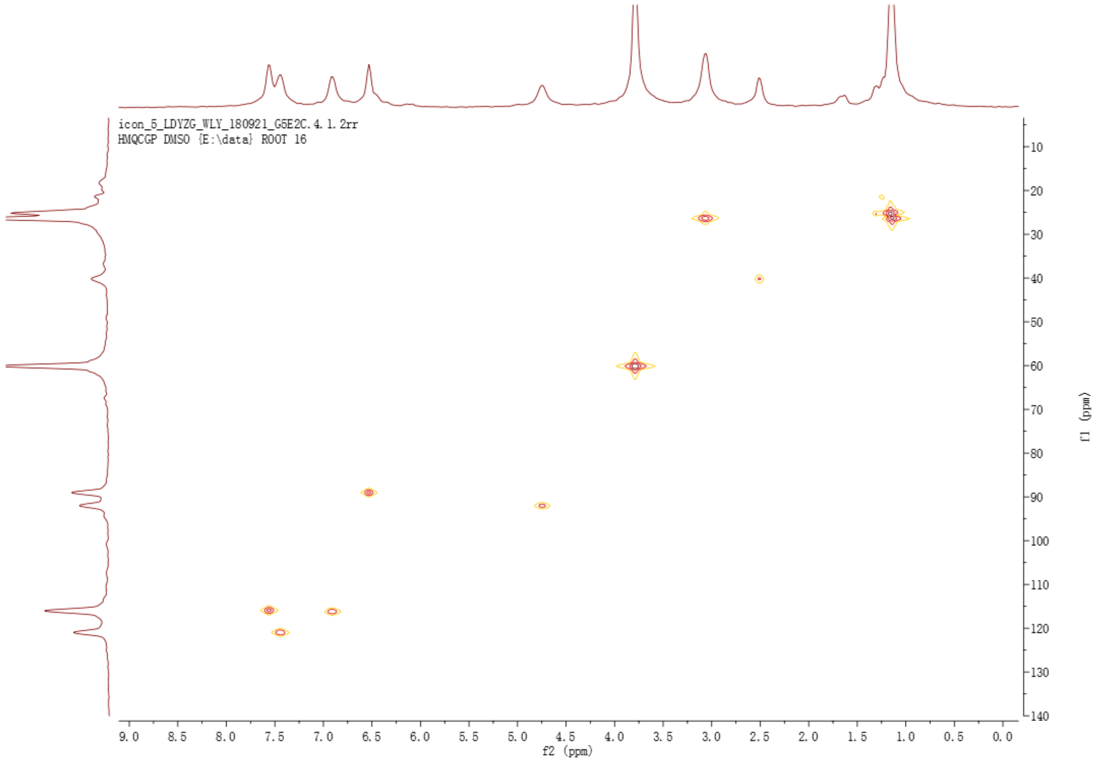

Figure S34: HSQC spectrum of compound **4**

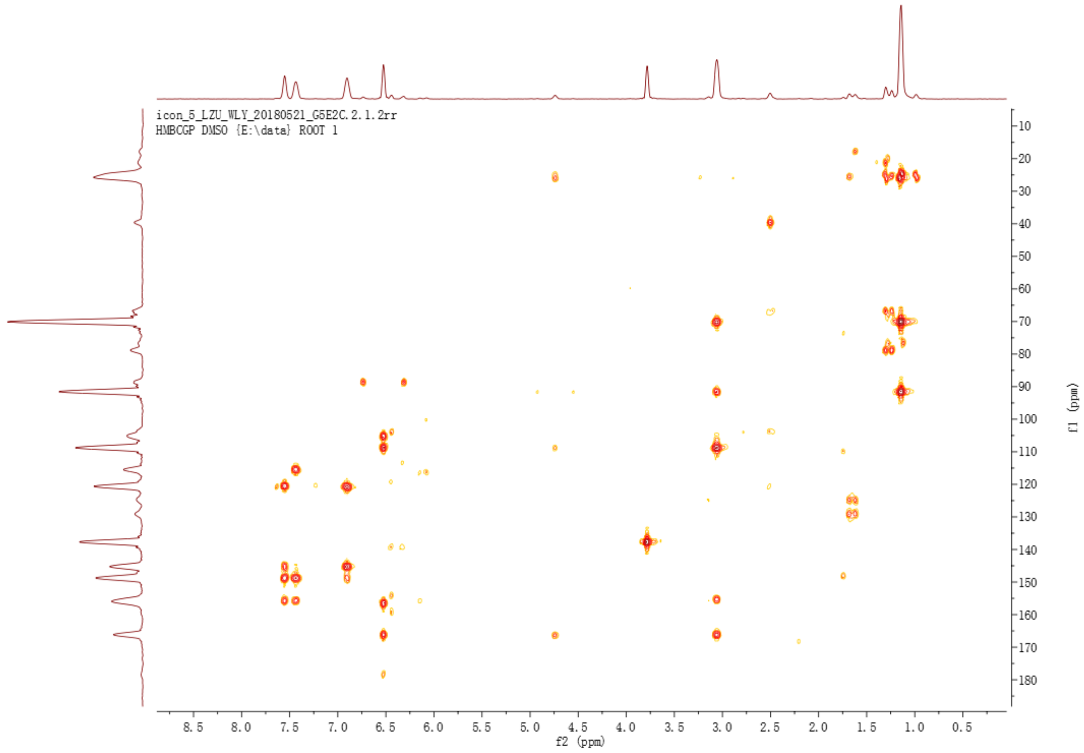

Figure S35: HMBC spectrum of compound **4**

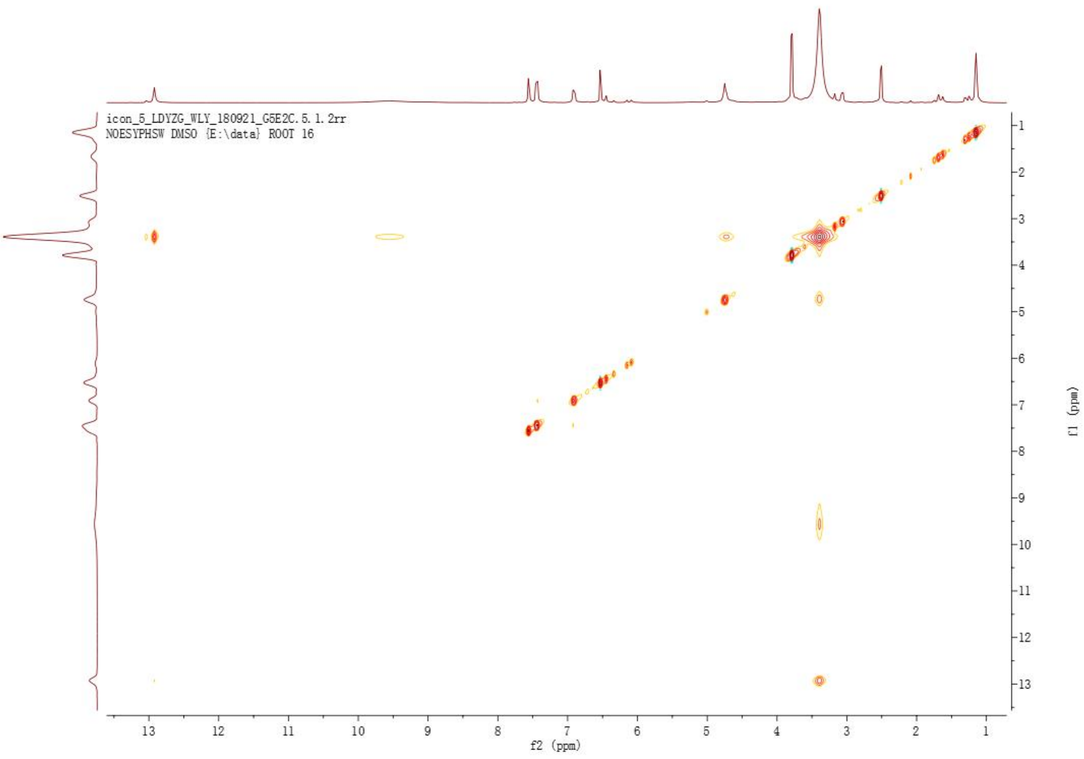

Figure S36: NOESY spectrum of compound **4**

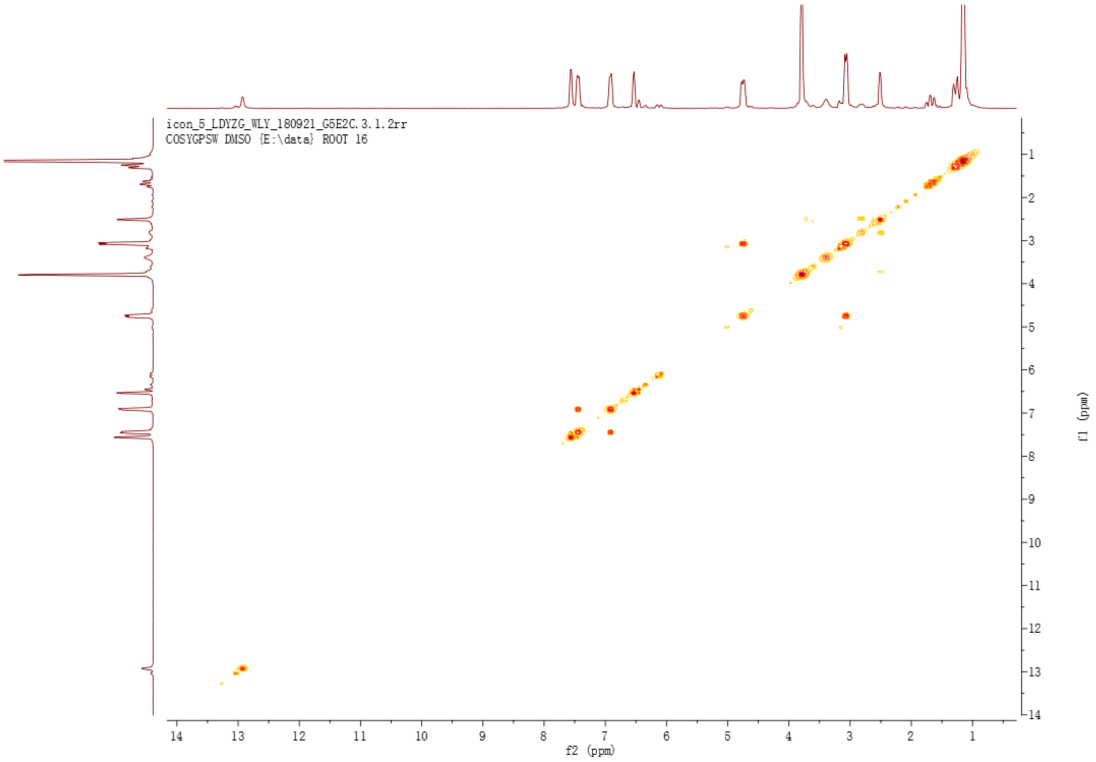

Figure S37: COSY spectrum of compound **4**

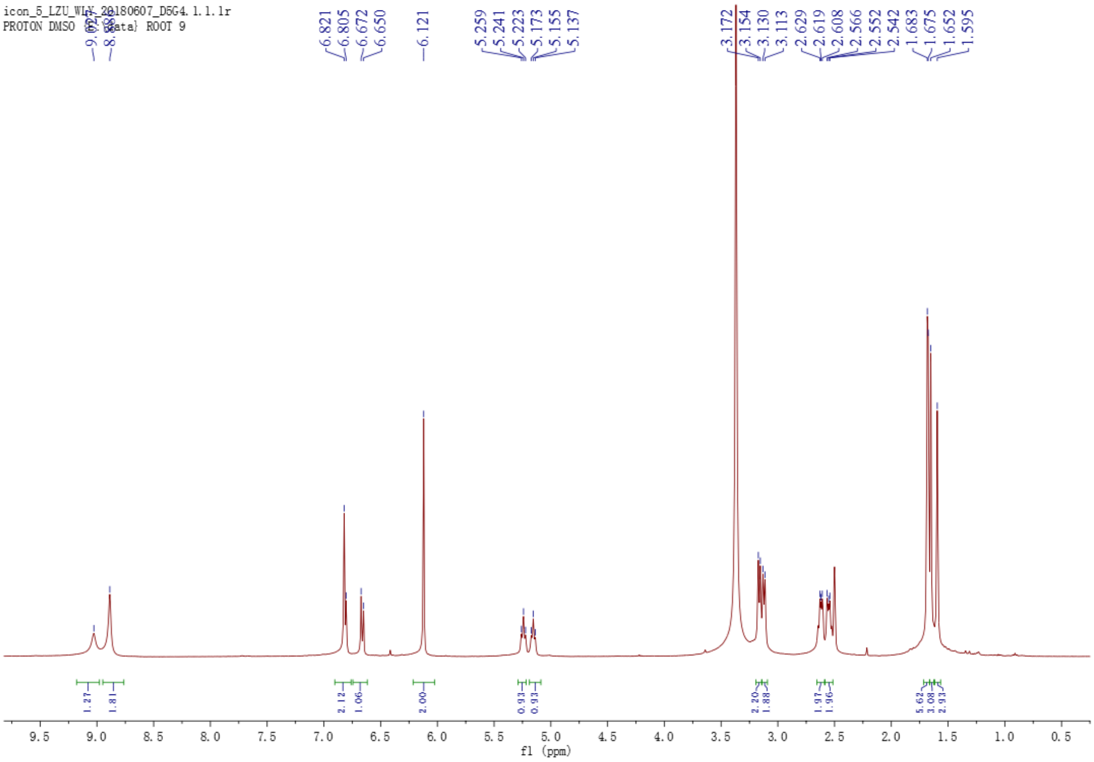

Figure S38: ^{1}H-NMR spectrum of compound **5**

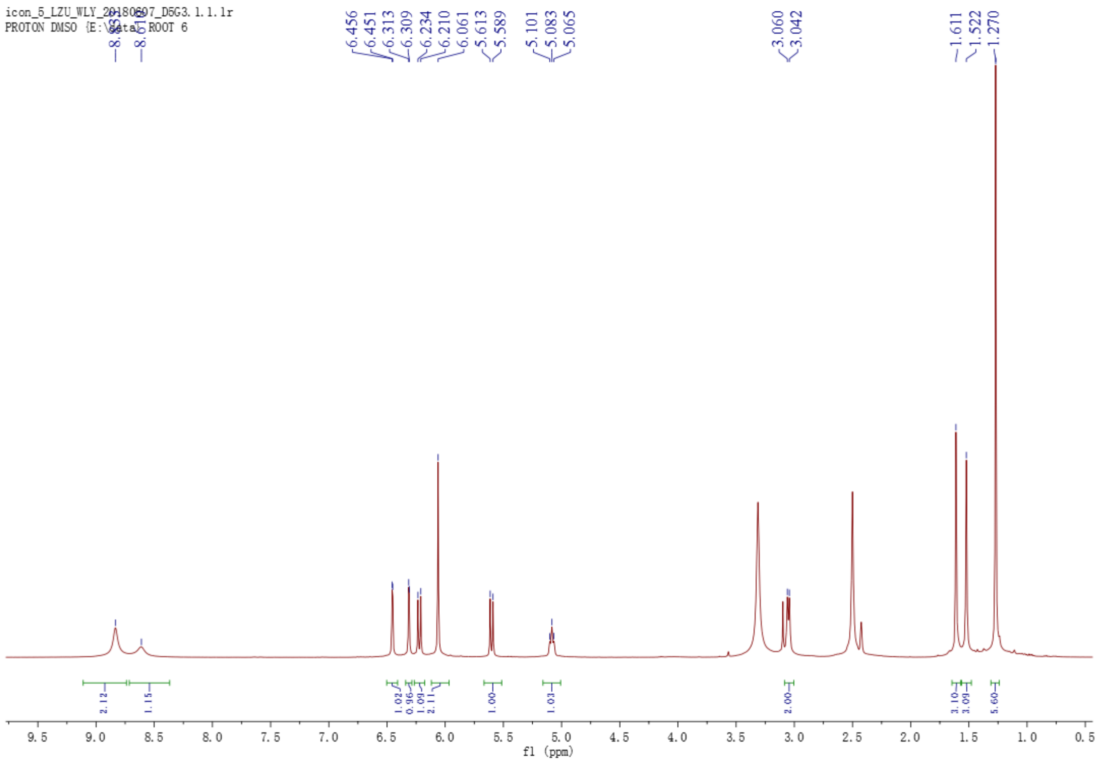

Figure S39: ^{1}H-NMR spectrum of compound **6**

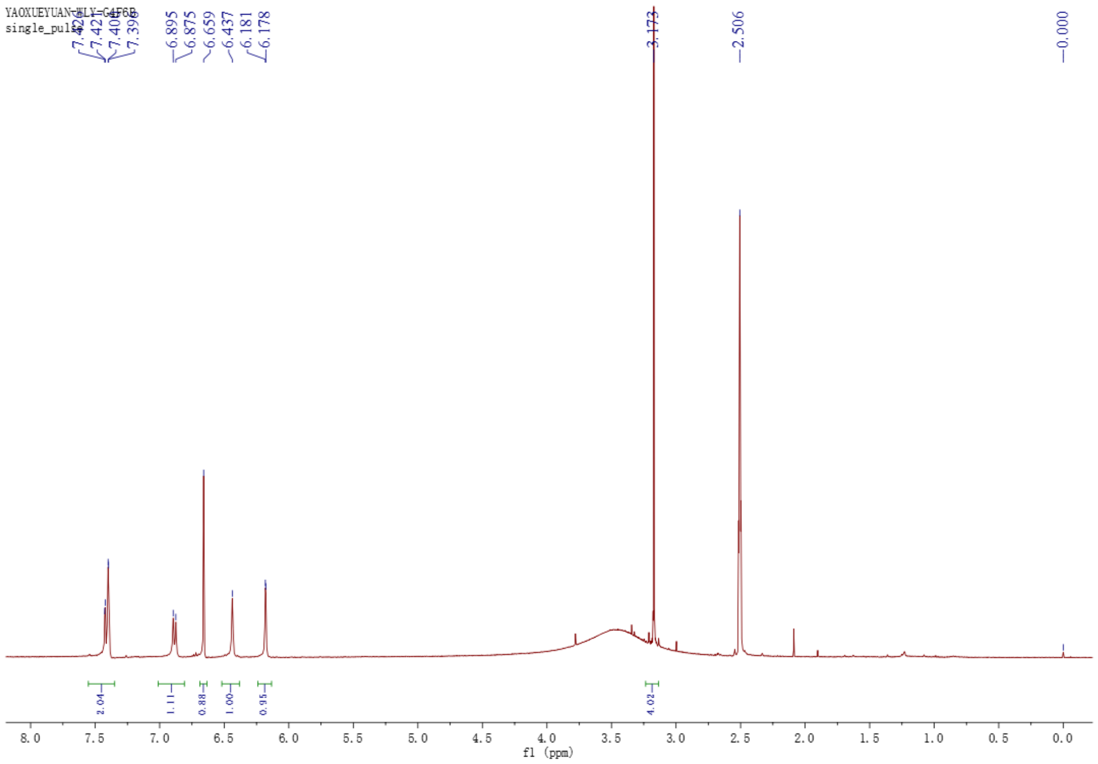

Figure S40: ^{1}H-NMR spectrum of compound **7**

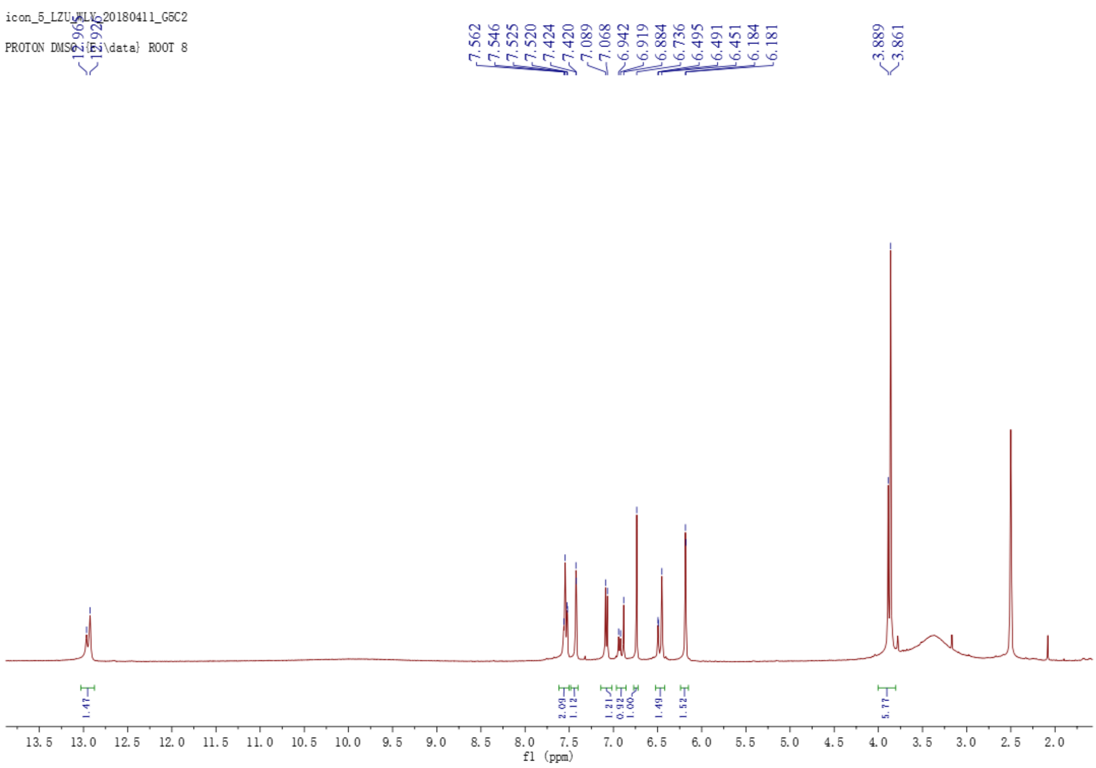

Figure S41: ^{1}H-NMR spectrum of compound **8**

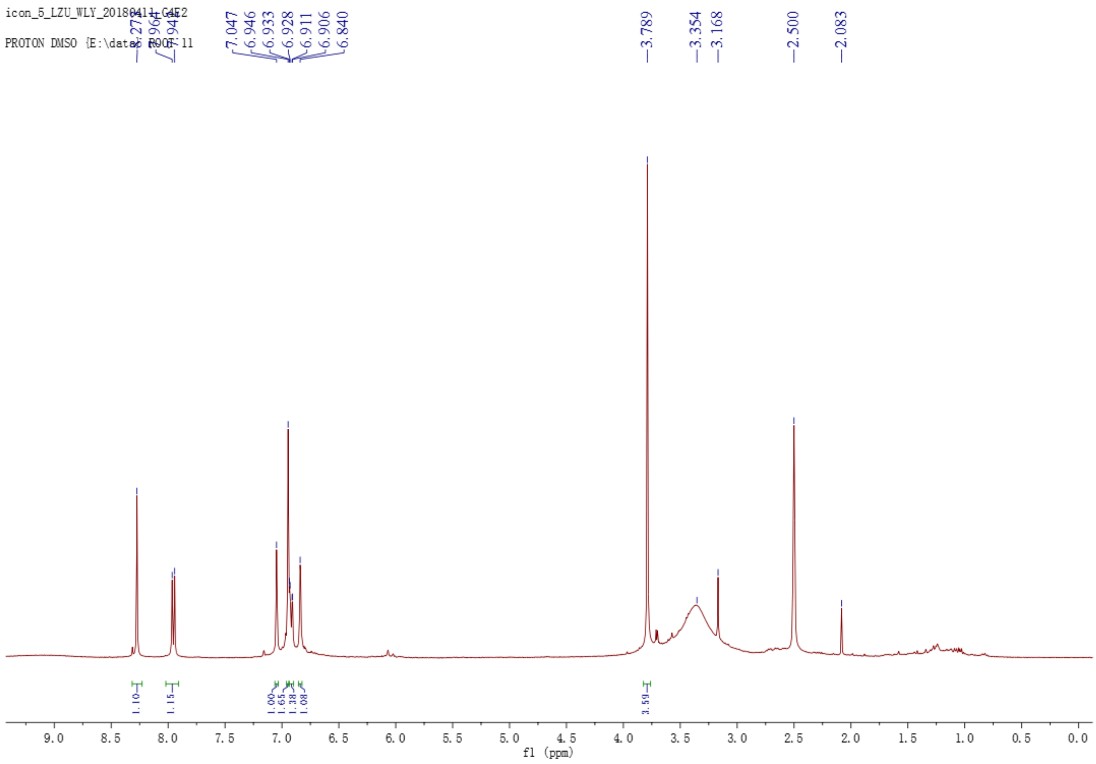

Figure S42: ^{1}H-NMR spectrum of compound **9**

Table S1: The % composition of compounds **1-12** in the leaves of *G. uralensis*

**compound**	**weight(mg)**	**total weight of leaves(kg)**	**composition(%)**
licostilbene A (**1**)	18.5	8	0.0002313
licostilbene B (**2**)	8.9	8	0.0001113
licofuranol A (**3**)	26.5	8	0.0003313
licofuranol B (**4**)	15.8	8	0.0001975
α,α'-dihydro-3,5,4′-trihydroxy-4,5′-diisopentenylstilbene (**5**)	12.6	8	0.0001575
glycypytilbene B (**6**)	28.4	8	0.0003550
Luteolin (**7**)	5.1	8	0.0000638
quercetin-3,4′-dimethyl ether (**8**)	4.2	8	0.0000525
calycosin (**9**)	6.7	8	0.0000838
scopoletin (**10**)	2	8	0.0000250
diosmetin (**11**)	7.9	8	0.0000988
echinatin (**12**)	4.6	8	0.0000575
